# Structured and disordered regions of Ataxin-2 contribute differently to the specificity and efficiency of mRNP granule formation

**DOI:** 10.1371/journal.pgen.1011251

**Published:** 2024-05-20

**Authors:** Arnas Petrauskas, Daniel L. Fortunati, Arvind Reddy Kandi, Sai Shruti Pothapragada, Khushboo Agrawal, Amanjot Singh, Joern Huelsmeier, Jens Hillebrand, Georgia Brown, Dhananjay Chaturvedi, Jongbo Lee, Chunghun Lim, Georg Auburger, K. VijayRaghavan, Mani Ramaswami, Baskar Bakthavachalu

**Affiliations:** 1 Trinity College Institute of Neuroscience, School of Genetics and Microbiology, Smurfit Institute of Genetics and School of Natural Sciences, Trinity College Dublin, Dublin, Ireland; 2 School of Biosciences and Bioengineering, Indian Institute of Technology, Mandi, India; 3 National Centre for Biological Sciences, TIFR, Bangalore, India; 4 Tata Institute for Genetics and Society Centre at inStem, Bellary Road, Bangalore, India; 5 School of Biotechnology, Amrita Vishwa Vidyapeetham University, Kollam, Kerala, India; 6 Manipal Institute of Regenerative Medicine, MAHE-Bengaluru, Govindapura, Bengaluru, India; 7 Department of Biological Sciences, Ulsan National Institute of Science and Technology (UNIST), 50 UNIST-gil, Ulsan, Republic of Korea; 8 Experimental Neurology, Medical School, Goethe University, Frankfurt, Germany; Geisel School of Medicine at Dartmouth, UNITED STATES

## Abstract

Ataxin-2 (*ATXN2*) is a gene implicated in spinocerebellar ataxia type II (SCA2), amyotrophic lateral sclerosis (ALS) and Parkinsonism. The encoded protein is a therapeutic target for ALS and related conditions. ATXN2 (or Atx2 in insects) can function in translational activation, translational repression, mRNA stability and in the assembly of mRNP-granules, a process mediated by intrinsically disordered regions (IDRs). Previous work has shown that the LSm (Like-Sm) domain of Atx2, which can help stimulate mRNA translation, antagonizes mRNP-granule assembly. Here we advance these findings through a series of experiments on *Drosophila* and human Ataxin-2 proteins. Results of Targets of RNA Binding Proteins Identified by Editing (TRIBE), co-localization and immunoprecipitation experiments indicate that a polyA-binding protein (PABP) interacting, PAM2 motif of Ataxin-2 may be a major determinant of the mRNA and protein content of Ataxin-2 mRNP granules. Experiments with transgenic *Drosophila* indicate that while the Atx2-LSm domain may protect against neurodegeneration, structured PAM2- and unstructured IDR- interactions both support Atx2-induced cytotoxicity. Taken together, the data lead to a proposal for how Ataxin-2 interactions are remodelled during translational control and how structured and non-structured interactions contribute differently to the specificity and efficiency of RNP granule condensation as well as to neurodegeneration.

## Author summary

Mutations in genes that promote protein aggregation are primarily associated with neurodegenerative diseases. RNA-binding proteins, crucial for gene regulation and cellular stress response, are also major constituents of protein aggregate-rich mRNP granules. Recent studies aim to understand the molecular mechanisms governing the formation of stress granules identified Intrinsically Disordered Regions (IDRs) within mRNP granule proteins as a key factor that facilitates the formation of amyloid-like structures. A deeper understanding of stress granule dynamics holds promise for elucidating neurodegenerative disease mechanisms. Ataxin-2, is an RNA-binding protein implicated in neurodegenerative disorders like spinocerebellar ataxia type-2, amyotrophic lateral sclerosis, and Parkinsonism. Reducing the levels of Ataxin-2 or deleting its IDR prevents RNP aggregation and offers neuroprotection in animal models of neurodegenerative diseases. We show that deleting the structured LSm domain of Ataxin-2, which impedes granule assembly, enhances cytotoxicity. Moreover, specific molecular interactions involving Ataxin-2 PAM2 motif binding to polyA-binding protein is crucial for recruiting mRNAs and other proteins into RNP granules. These interactions not only shape granule composition but also influence disease progression. Our findings propose a model illustrating the dynamic remodeling of Ataxin-2 interactions, emphasizing the distinct roles of structured and unstructured domains in governing mRNP-granule condensation and neurodegeneration.

## Introduction

mRNP granules are intriguing, dynamic membrane-less organelles containing translationally repressed mRNAs, RNA-binding proteins (RBPs), molecular chaperones and a variety of other cellular proteins [1–5]. The formation and composition of mRNP assemblies are determined by base-pairing interactions between mRNAs, protein-protein interactions and RBP-RNA interactions, whose respective contributions may vary across granule types and physiological states [6–9]. Stress granules (SGs) are particularly well-studied granules that form when cellular stress signals mediated by eIF2α kinase activation [10] cause individual mRNPs to arrest in translation and condense into multi-mRNP assemblies [11–13]. Mutations in mRNP granule proteins, including TDP-43, FUS, Ataxin-2, hnRNPA1, hnRNPA2B1, EWSR1, have been associated with ALS and/or other forms of neurodegenerative disease [14–20]. For this reason, and because TDP-43 and other stress-granule protein aggregates are components of protein inclusions found in ALS and Frontotemporal dementia (FTD), the regulation and cellular functions of SGs have been topics of considerable fundamental and clinical interest [20–26].

The cast of intermolecular interactions required for mRNP-granule assembly and the precise sequence with which they occur are not yet elucidated [9,27]. However, many studies show that intrinsically disordered regions (IDRs) found on mRNP-granule proteins contribute substantially to RNP granule assembly [28–35]. In biochemical experiments, such IDRs show the ability to phase separate into liquid-like assemblies [35–45]. The accessibility or activities of IDRs can be tightly regulated by posttranslational modifications, allowing rapid physiological and spatial control over granule assembly and disassembly [29,35,46–52]. An important observation is that most IDRs also can transition from liquid-like state into solid, beta-sheet rich, amyloid-fibrils *in vitro*, particularly at high concentrations achieved in the liquid phase [22,41,53–55]. This, and studies showing that inhibitors of eIF2α kinase or downstream events including SG formation can be protective in animal models of neurodegenerative disease [56–60] have led to a conceptual framework in which: (a) mRNP granules provide a microenvironment where pathogenic protein seeds can form and grow [54,61,62]; (b) increased misfolded protein loads result in inclusion formation, chronic stress signalling and reduced protein translation [63,64]; (c) increased demand on protein handling systems results in multiple cellular defects, notably in the functions of membrane-less organelles [65–69]. In particular, aberrant SG formation also results in nuclear transport defects which may contribute to cell death and toxicity [70,71].

Particularly strong support for the role of RNP granule formation in promoting disease comes from studies of Ataxin-2. Loss of Ataxin-2 is cytoprotective in yeast TDP-43 and *Drosophila* TDP-43 or C9ORF72 or Tau models of cytotoxicity [16,61,72–76]. In mouse models for SCA2 or ALS, either genetic loss of *ATXN2* or delivery of antisense oligonucleotides (ASOs) targeting *ATXN2* in the central nervous system, reduced aggregation of TDP-43, increased animal survival and improved motor function [72,77]. These observations have led to ASOs against human *ATXN2* being developed and approved for clinical trials [78]. More recent genome-wide screens using siRNA and CRISPR have identified additional strategies to lower the levels of Ataxin-2 [79,80].

Given Ataxin-2’s therapeutic significance and multiple roles in biology, it is particularly important to determine which molecular activities of the protein are relevant to disease and to its various biological functions [81]. Across species, Ataxin-2 has three conserved regions: a Like-Sm (LSm) domain, an LSm-associated domain (LSm-AD) and a PAM2 motif, which is flanked by extended IDRs [82]. Detailed work in *Drosophila* has shown that a c-terminal IDR of Atx2 is selectively required for mRNP assembly into granules [61]. Parallel experiments showing that the IDR is also required for cytotoxicity in *Drosophila* FUS, C9ORF72 and Huntington’s disease models suggest RNP-granule formation to be a significant mechanism by which Atx2 promotes neurodegeneration [61,74]. A recent discovery that the Atx2-LSm domain antagonizes IDR-function has led to a model in which the Atx2 cIDR: (a) does not support mRNP assembly when Atx2 is associated with actively translating mRNAs through Atx2-LSm domain interactions; (b) becomes accessible and active in mediating mRNP assembly when LSm-domain interactions break and mRNA translation stalls [82,83].

We now present a series of experiments further elaborating mechanisms by which Ataxin-2 functions in mRNA translation and mRNP assembly. These show that the PAM2 motif of Ataxin-2 and its interactions with PABP are not essential for granule assembly but are required to efficiently recruit Atx2-target mRNAs and specific protein components into Ataxin-2 granules. When taken together with other findings [73,82–84], our observations indicate that PAM2 binding to PABP on the polyA tail of mRNAs helps specify the composition of Ataxin-2 granules. We propose an early role for PAM2:PABP interactions working in coordination with the LSm domain to support mRNA translation and thereby oppose the mRNP formation [82]; as well as a later role in escorting translationally-stalled PAM2:PABP linked mRNAs into mRNP granules. *In vivo* experiments analysing motor decline in transgenic *Drosophila* indicate that the PAM2:PABP interactions also support the progression of the neurodegenerative process. We provide new evidence for fresh insight into the enigmatic role of mRNP assembly in neurodegeneration.

## Results

### The structured PAM2 domain of Atx2 is necessary for the correct mRNA and protein content of Atx2 granules

A recent work from our lab used Targets of RNA-Binding Proteins Identified by Editing (TRIBE) technology to identify mRNAs associated with Atx2 in the *Drosophila* adult brain [83]. *In vivo*, the ability of an Atx2-fusion with ADARcd (the catalytic domain of an RNA-editing enzyme, ADAR), to edit a group of 256 target mRNAs was found to be dependent on the presence of the Atx2-cIDR, previously shown to be necessary for the formation of neuronal mRNP granules *in vivo*. In contrast, Atx2-ADARcd mutants lacking the LSm domain, both edited Atx2 TRIBE target RNAs and formed mRNP granules in cultured *Drosophila* S2 cells more efficiently than the wild-type. Thus, Atx2-ADARcd editing of target mRNAs occurs in and is reflective of mRNP granule assembly. While demonstrating a role for LSm-domain interactions in antagonizing cIDR-mediated granule assembly, these observations did not address mechanisms by which Atx2 target mRNAs are selected, or whether and how Atx2 played any role in determining the composition of RNP granules. New experiments presented here address these outstanding questions.

Previous TRIBE analyses showed that LSm and LSm-AD regions have no major role in the recognition or selection of the Atx2-target mRNAs [83]. We therefore tested whether the third conserved region of Ataxin-2, a PAM2 motif known to associate with PABP (polyA binding protein), played any role in this process [85,86].

**Fig 1:**
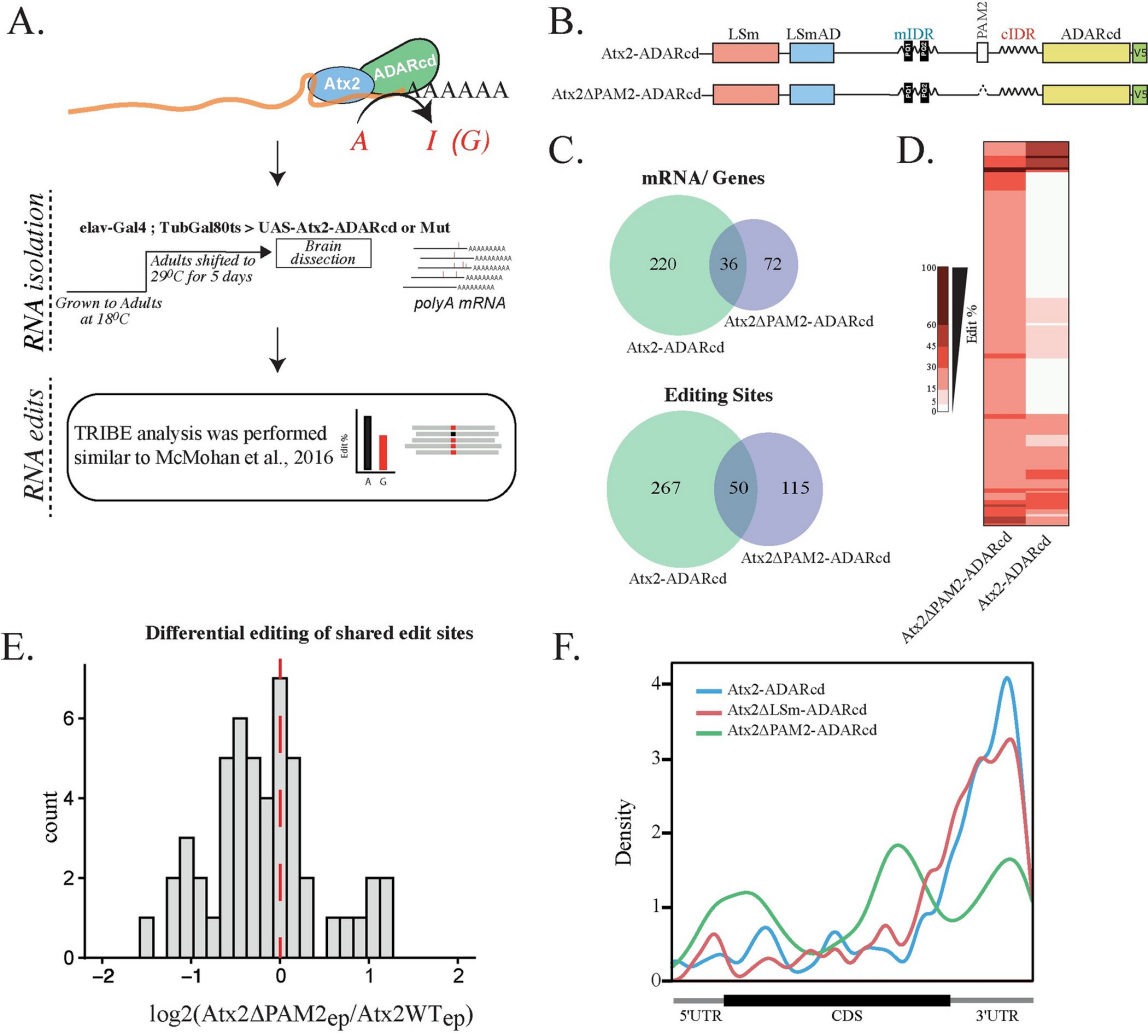
The PAM2 domain facilitates the selection of Atx2 RNA targets. **(**A). The flowchart illustrates the TRIBE analysis pipeline. Atx2ΔPAM2-ADARcd was expressed in adult *Drosophila* brains using the Gal4-responsive UAS-Atx2 transgene, under the control of elav-Gal4 and TubGal80ts. The temperature-sensitive TubGal80ts enables us to induce the expression of the UAS-Atx2 transgene selectively in the adult fly brains by shifting the temperature from 18°C to 29°C. Total brain RNA was isolated and RNA edits were identified and compared to Atx2-ADARcd, similar to Singh et al 2021[83]. (B) Domain map of Atx2-ADARcd constructs used for TRIBE analysis. (C) Comparisons of genes and edits identified by TRIBE between Atx2-ADARcd and Atx2ΔPAM2-ADARcd targets. (D) Most Atx2ΔPAM2 targets identified by TRIBE are unique and not edited in Atx2WT, suggesting that these new targets bound by Atx2ΔPAM2 are not native Ataxin-2 granule targets. (E) Comparisons of the editing efficiency ratio of common edits between Atx2WT vs Atx2ΔPAM2 show a much lower editing efficiency in Atx2ΔPAM2 compared to Atx2WT. This is despite comparable expression levels of the Atx2 fusion protein forms in brains (panel A of I Fig in S1 File). (F) PAM2 deletion results in loss of 3’UTR specificity seen in Atx2WT and LSm deletion TRIBE target mRNAs. Atx2WT and Atx2ΔLSm-ADARcd data are extracted from [83]. Controls and comparisons of replicates are summarised in I Fig in S1 File.

We used Gal80^ts^-controlled *elav-Gal4* to express Atx2ΔPAM2-ADARcd (deleted for the PAM2 motif) in brains of adult *Drosophila* for 5 days and used RNA-Seq to identify edited RNAs in polyA selected brain RNA and compared it with Atx2-ADARcd using procedures described earlier (Fig 1A) [87]. ADAR-edits, which convert Adenosine to Inosine on RNAs, are identified as A to G changes in TRIBE analyses. Each sample was sequenced to obtain 20 million reads (Table A in S1 File). The edits were only considered from the regions of the transcriptome that contained at least 20 reads. Genes with edits identified at a threshold above 15% in two biological replicates were considered as high confidence true targets. We compared edit frequency and edited gene identity in the brains of flies expressing Atx2ΔPAM2-ADARcd with those in brains expressing Atx2-ADARcd.

In contrast to Atx2 forms lacking LSm or LSm-AD domains [83], Atx2ΔPAM2-ADARcd edited significantly fewer RNA targets than wild-type Atx2-ADARcd (108 genes and 165 edits vs 256 genes and 317 edits, Fig 1B and 1C, and Table B in S1 File). This is despite comparable protein and RNA expression levels (panels A and B of I Fig in S1 File). More striking, the cohort of mRNAs edited by the ΔPAM2 mutant form differed extensively from the largely overlapping cohorts edited by either wild-type forms of Atx2 (panels C and D of I Fig in S1 File). Of the 108 genes edited by Atx2ΔPAM2-ADARcd, 36 were also targets of Atx2-ADARcd, the remaining 72 were unique. (Fig 1C and 1D, and Table B in S1 File). Fifty edit sites were common between the Atx2ΔPAM2 and Atx2WT targets. Those sites were edited with much lower efficiency in Atx2ΔPAM2 as compared to Atx2WT (Fig 1E).

The location of the edits made by Atx2ΔPAM2-ADARcd also differed dramatically as to where they occurred relative to the coding sequences of the target mRNAs (Fig 1F). While edits made by wild-type and ΔLSm forms of Atx2-ADARcd were greatly enriched in the 3’UTR of the mRNAs, Atx2ΔPAM2 targets were edited indiscriminately all along the mRNA length (Fig 1F).

Taken together, these data identify the PAM2 motif as necessary for Atx2 engagement with its correct mRNA targets. The PAM2 motif interacts with PABP, which binds polyA tracts at the 3’ end of mRNAs [88]. The distribution of targets across the expression level spectrum of all sequenced mRNAs further supports the role of the PAM2 domain in specifically associating with a subset of mRNAs (panel C of I Fig in S1 File). Therefore, the data point to a role for the structured PAM2:PABP interaction in guiding the association of Atx2 with mRNAs and for the subsequent inclusion of these mRNAs in Atx2-containing granules.

**Fig 2:**
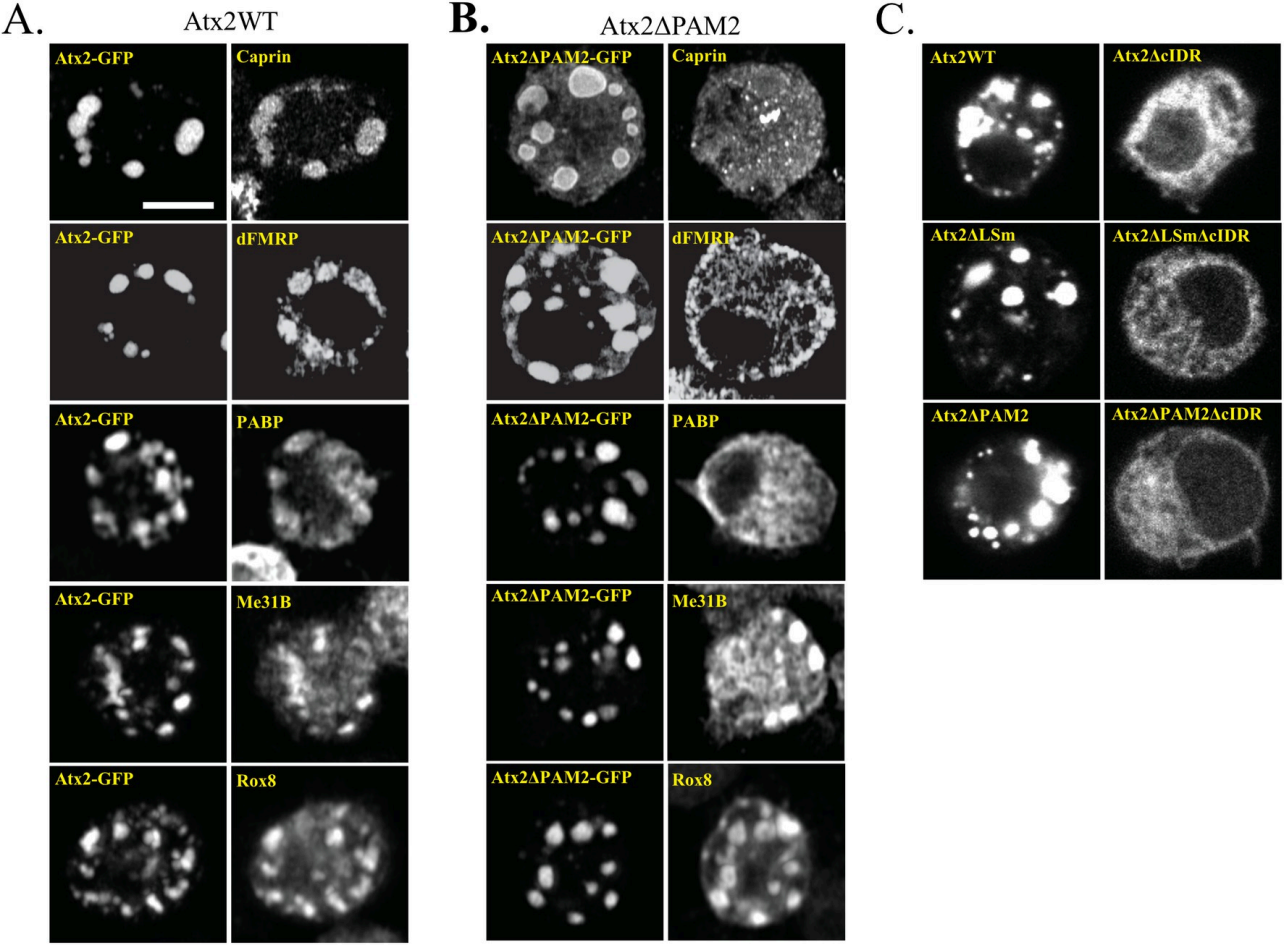
The presence of the PAM2 domain affects the protein composition of Atx2-GFP granules in S2 cells. (A) Over-expression of Atx2-GFP in unstressed *Drosophila* S2 cells induces the formation of Atx2-GFP granules to which various SG markers, namely, Caprin, dFMRP, PABP, Me31B and Rox8, co-localize. (B) Deletion of the PAM2 affects the Atx2-GFP granule composition. Over-expression of Atx2ΔPAM2-GFP in S2 cells still induces the formation of granules, but some SG markers fail to co-localize in these, notably dFMR, Caprin and PABP. In contrast, co-localisation with dFMR, Caprin and PABP is not affected by the deletion of the LSm domain in analogous experiments (III Fig in S1 File). (C) Atx2-GFP granule formation in S2 cells relies primarily on the cIDR. Deletion of the cIDR in Atx2WT, Atx2ΔPAM2 and Atx2ΔLSm, removes their ability to form granules upon overexpression. See panels A-B of II Fig in S1 File, for quantification. The scale bar in (A) applies to (B) and (C). Scale bar = 5 μm.

If Atx2-ADARcd edits of target mRNAs occur predominantly in the mRNP granules [83], then the ability of Atx2ΔPAM2-ADARcd to edit some target mRNAs would suggest that the PAM2 motif is not essential for mRNP granule formation *per se*. To examine this, we expressed wild-type and ΔPAM2 mutant forms of GFP-tagged Atx2 under the control of a native genomic promoter in *Drosophila* S2R+ cells. Atx2 overexpression in S2 cells induced the formation of mRNP granules closely related to SGs, containing endogenous Atx2 and various SG proteins as previously reported (Fig 2A) [61,83]. Similar expression of Atx2ΔPAM2-GFP also induced granule formation. However, these granules were compositionally distinct from those induced by Atx2-GFP. While they contained some SG markers present on Atx2-granules, e.g., Me31B and Rox8 (*Drosophila* homologs of DDX6 and TIA1), they did not contain others such as PABP, Caprin and dFMRP that are present in both wild-type Atx2-GFP and Atx2ΔLSm-GFP granules (Fig 2B, and III Fig in S1 File).

RNP-granules induced by expression of wild-type, LSm and PAM2 deficient forms of Atx2-GFP required the presence of the c-terminal IDR (Fig 2C). Thus, while largely dispensable for efficient mRNP assembly, the PAM2 domain plays a significant role in determining both mRNA and protein components of mRNP granules. One possibility is that the PAM2 motif directly recruits PABP and associated mRNAs to granules and indirectly recruits other proteins through their interactions with either PABP or mRNAs brought to RNP granules through Atx2-PAM2:PABP interactions.

### PAM2:PABP interactions are sufficient for Atx2 to associate with stress granules

**Fig 3:**
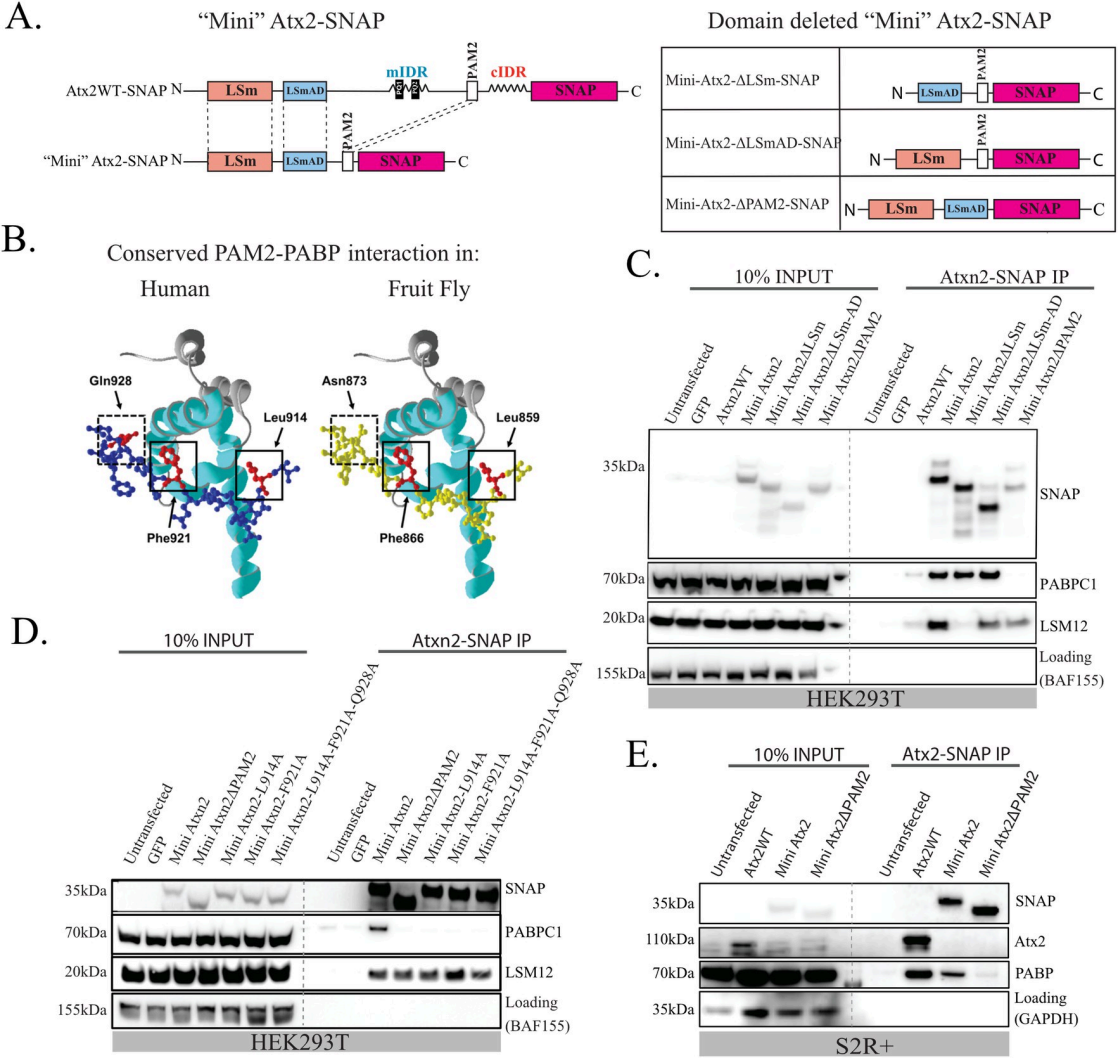
A minimized Ataxin-2 construct containing only the known structured domains maintains the ability to interact with PABP and LSM12. (A) Schematic of SNAP-tagged full length, minimal, and minimal domain-deleted constructs of fly Atx2 used to isolate the function of the structured domains of the protein without interference from IDR-mediated interactions. Human and *Drosophila* Ataxin-2 LSm, LSmAD and PAM2 domains show high amino acid sequence similarity (Clustal Ω) of 70%, 82% and 87% respectively. This suggests a conserved and specific function of these structured domains. (B) Structural model of the PABP MLLE domain (ribbon) showing the near-perfect structural similarity of the human ATXN2 PAM2 domain (blue, uniprot ID: Q99700) with the *Drosophila* Atx2 PAM2 domain (yellow, uniprot ID: Q8SWR8). The key interacting residues are highlighted in red. (C) Minimal human ATXN2 SNAP IP-WB from HEK293T cells probing for PABP and LSM12 showing the effects of different domain deletions. The PAM2 domain is necessary and sufficient for the ATXN2-PABP interaction, while the LSm domain is necessary and sufficient for the ATXN2-LSM12 interaction. (D) Point mutations targeting key interacting residues of the PAM2 domain were predicted to replicate the effect of a full PAM2 deletion in the minimized Atx2 construct. Human minimized ATXN2-SNAP IP WB from HEK293T cells showing that mutating either of the key hydrophobic residues L914 or F921 in the PAM2 domain is sufficient to prevent its interaction with PABP. The interaction with LSM12 is unaffected by the point mutations. (E) *Drosophila* minimized Atx2-SNAP IP-WB from S2 cells. An analogous PAM2 domain deletion blocks the Atx2-PABP interaction and pull-down in experiments. The conserved amino acid point mutation on F866 similarly blocks the Atx2-PABP interaction (panels D-E of IV Fig in S1 File). Replicates and controls for IP-WB experiments are shown in panels A-E of IV Fig in S1 File.

**Fig 4:**
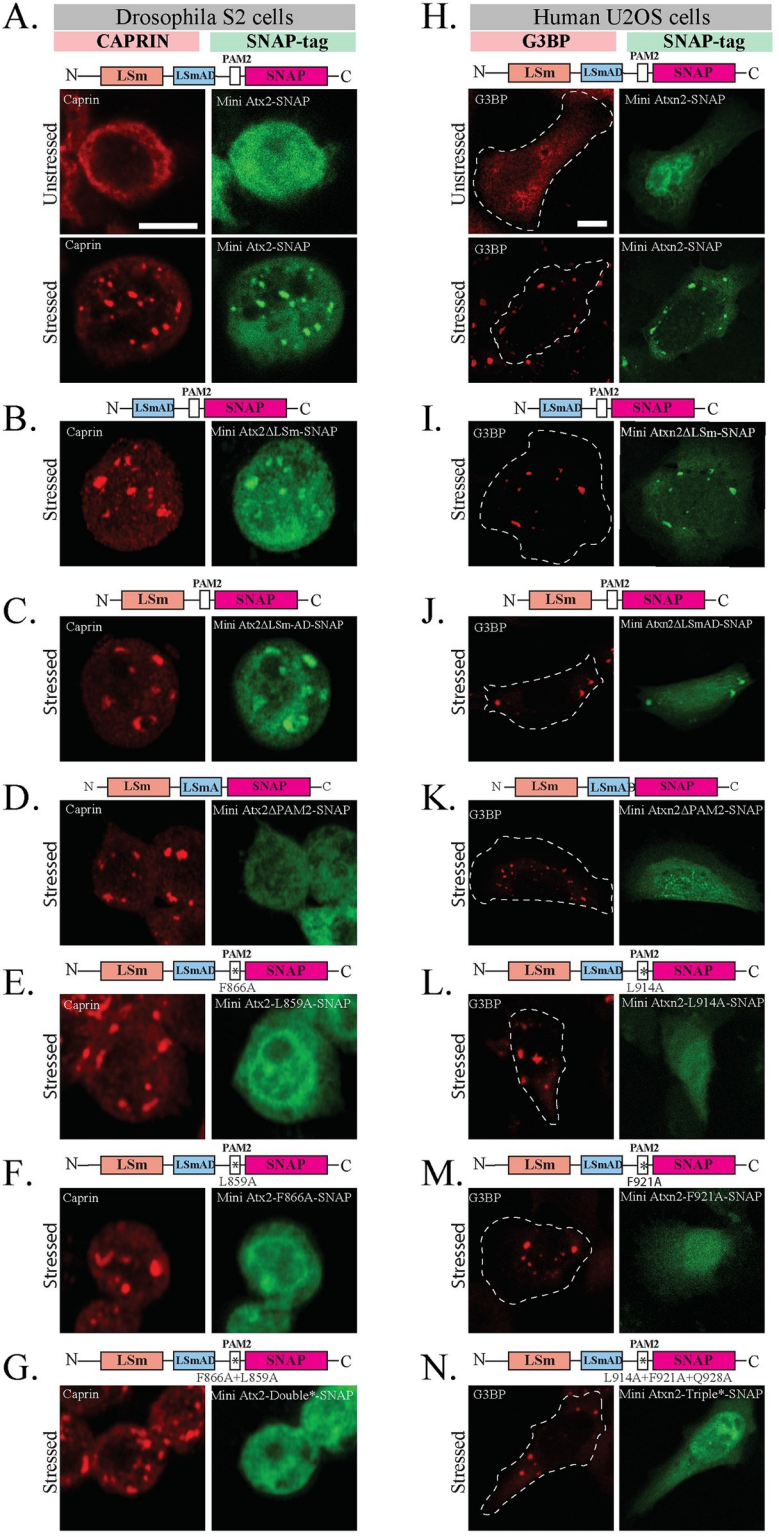
The structured PAM2 domain is necessary and sufficient for Ataxin-2 recruitment to Stress Granules in both *Drosophila* and human cells. (A) In *Drosophila* S2 cells mini-Atx2-SNAP (green) is recruited to SGs induced by arsenite (red). (B) Deletion of LSm or (C) LSm-AD domains has no significant effect on the arsenite-induced SG recruitment. (D) The presence of the PAM2 domain, and specifically its key PABP-interacting amino acids (E-G), is necessary for Atx2 recruitment to SGs. Caprin (red) was used as SG granule marker, scale bar = 5 μm, (H) Human mini-ATXN2-SNAP (green) is recruited to arsenite-induced SGs in human U2OS cells. (I) Deletion of LSm or (J) LSm-AD domains does not affect the arsenite-induced SG recruitment of ATXN2. (K) Deletion of the PAM2 domain, and specifically its PABP-interacting amino acids (L-N), are necessary for ATXN2 recruitment to SGs. G3BP1 (red) was used as SG marker; scale bar = 10 μm. The schematics above images indicate the domain deletions or amino acid mutations that were made in the different Ataxin-2 constructs. See panel C of II Fig in S1 File for quantification.

We wanted to directly confirm Ataxin-2 PAM2 motif interactions with PABP and analyse their relevance to RNP granule assembly. For this, we generated constructs encoding SNAP-epitope tagged variants of Atx2. These are radically truncated forms of *Drosophila* and human Ataxin-2 proteins containing only the LSm, LSm-AD and PAM2 elements and lacking all unstructured regions of the protein. The structured elements are connected via flexible linkers (Fig 3A). These “Mini-Ataxin-2” constructs and their domain-deleted forms allowed us to separate functions of the structured regions of Ataxin-2 from those of the remaining extended disordered regions. A similar approach has been previously shown for MeCP2 [89]. We identified key residues involved in *Drosophila* Atx2-PAM2:PABP interactions based on a previously solved crystal structure of a strongly conserved mammalian PAM2:PABPC1-MLLE domain complex [90,91] (Fig 3B). Residues leucine 914 and phenylalanine 921 (L914 and F921) in the human ATXN2-PAM2 motif are predicted to contact the PABPC1-MLLE domain and of these, F921 has been shown to be required for the PABPC1-ATXN2 interaction [92]. These residues (L859 and F866, respectively) in Atx2 and MLLE in PABP are perfectly conserved in flies (panels E-F of IV Fig in S1 File). In order to allow more precise disruption of PAM2:PABP interactions and avoid potential unknown secondary effects of larger PAM2 motif deletions, we additionally generated mini-Ataxin-2 constructs where these PABP-contacting residues were singly or doubly altered to alanine. We used these constructs for co-immunoprecipitation (Fig 3) and co-localization (Fig 4) analyses to examine the contribution of PAM2:PABP/PABPC1 interactions in RNP-granule formation.

We expressed SNAP-tagged wild-type and mutant forms of mammalian and *Drosophila* mini-Ataxin-2 in HEK293T and S2 cells, respectively. The overexpression of Ataxin-2 did not affect the levels of endogenous RNP granule proteins dFMRP and PABP in S2 cells. However, in Rin Western blot analysis, two bands were observed, and the ratio between the larger and smaller bands was altered in transfected cells (panel A of IV Fig in S1 File). We tested which Ataxin-2 domains were required for SNAP substrate beads to successfully immunoprecipitate Ataxin-2 complexes containing PABPC1/PABP from cell lysates (Fig 3C–3E). Both LSM12 and PABPC1 proteins were co-immunoprecipitated with mammalian mini-ATXN2. However, PABPC1 coimmunoprecipitation was selectively lost when the PAM2 domain was deleted or if predicted PABP-contact residues in the PAM2 domain were mutated (Fig 3C and 3D). Similar to the human homolog, fly mini-Atx2-SNAP also required the presence of its PAM2 motif with both predicted contact residues intact for immunoprecipitation of PABP from *Drosophila* S2 cell lysates (Fig 3E and panels B-D of IV Fig in S1 File). Taken together, these data support a potential sequence of molecular events. In unstressed cells, PAM2 domain interaction with PABP helps position Ataxin-2 at the 3’-end of mRNAs while LSM-domain mediated association with LSM12 stimulates translation of these mRNAs; under stress conditions (or Ataxin-2 overexpression), translation is arrested and the cIDR domain is freed to promote promiscuous interactions that facilitate the formation of condensed RNP granules (see Discussion).

Single mRNAs usually associate with multiple PABP molecules because their polyA tails are considerably longer than the ~24 bases required for PABP binding [93]. Thus, in cells expressing endogenous and mini-Ataxin-2, mRNAs could have both forms associated with their polyA tails. In response to stress, mini-Ataxin-2 would be expected to move into SGs whose formation is facilitated by IDRs on endogenous Ataxin-2 associated with the common target RNAs. We examined this possibility in cells before and after oxidative stress.

*Drosophila* and human mini-Ataxin-2-SNAP, expressed in fly S2 or human U2OS cells respectively, were diffusely localized in the cytoplasm and neither induced the formation of Ataxin-2 foci. However, when cells were exposed to sodium arsenite to induce SG formation, SNAP-tagged mini-Atx2 (Fig 4A) and mini-ATXN2 (Fig 4H) were robustly recruited to SGs. Thus, while granule assembly requires the IDRs of Ataxin-2, association of the protein with mRNP-granule components may be achieved by structured domain interactions alone, independently of mRNP assembly into granules.

Further experiments examined which of the LSm, LSm-AD and/or PAM2 domains were necessary for mini-Ataxin-2 to associate with SGs. Mammalian and *Drosophila* mini-Ataxin-2 forms missing the LSm or LSm-AD domains could still be found in SGs (Fig 4B, 4C, 4I, and 4J). In contrast, mutants lacking the PAM2 domain remained cytoplasmic after stress in both S2 (Fig 4D) and U2OS cells (Fig 4K). Notably, point mutations in the Ataxin-2-PAM2 domain that specifically disrupt PAM2:PABP interaction similarly prevent localization to SGs (Fig 4E–4G and 4L–4N). Thus, interactions between Ataxin-2’s PAM2 domain and PABP appear important for the presence of Ataxin-2 in native mRNP granules, whose assembly is driven by the distinct (IDR) region of the protein (Fig 2C). The ability of otherwise full-length Ataxin-2 lacking PAM2 to form compositionally distinct mRNP assemblies (Fig 2B) suggests that PAM2:PABP binding also serves to limit non-physiological interactions by Ataxin-2 (See Discussion).

### The IDR and PAM2 domains promote and the LSm domain inhibits cytotoxicity in *Drosophila* neurodegeneration models

Three different Ataxin-2 domain deletions tested showed three distinct effects on mRNP granule assembly in S2 cells. IDR domain deletions prevents Ataxin-2 granule formation. LSm domain deletion enhances the formation of Ataxin-2 granules [82,83]. PAM2 domain deletions result in the formation of unusual mRNP assemblies (Fig 2B and 2C). Prior observations showing that Atx2 IDR deletions suppress cytotoxicity in *Drosophila* models for neurodegeneration indicate that mRNP granules support events that lead to degenerative disease [61,72,74,77]. If true, the expression of Atx2ΔLSm, which enhances granule assembly, would promote or potentially accelerate degeneration, while the expression of Atx2ΔcIDR would not. The expression of Atx2ΔPAM2 would be expected to support mRNP assemblies of different compositions from the ones containing wild-type or ΔLSm forms of Atx2. The effects on degeneration for this condition would be hard to predict.

**Fig 5:**
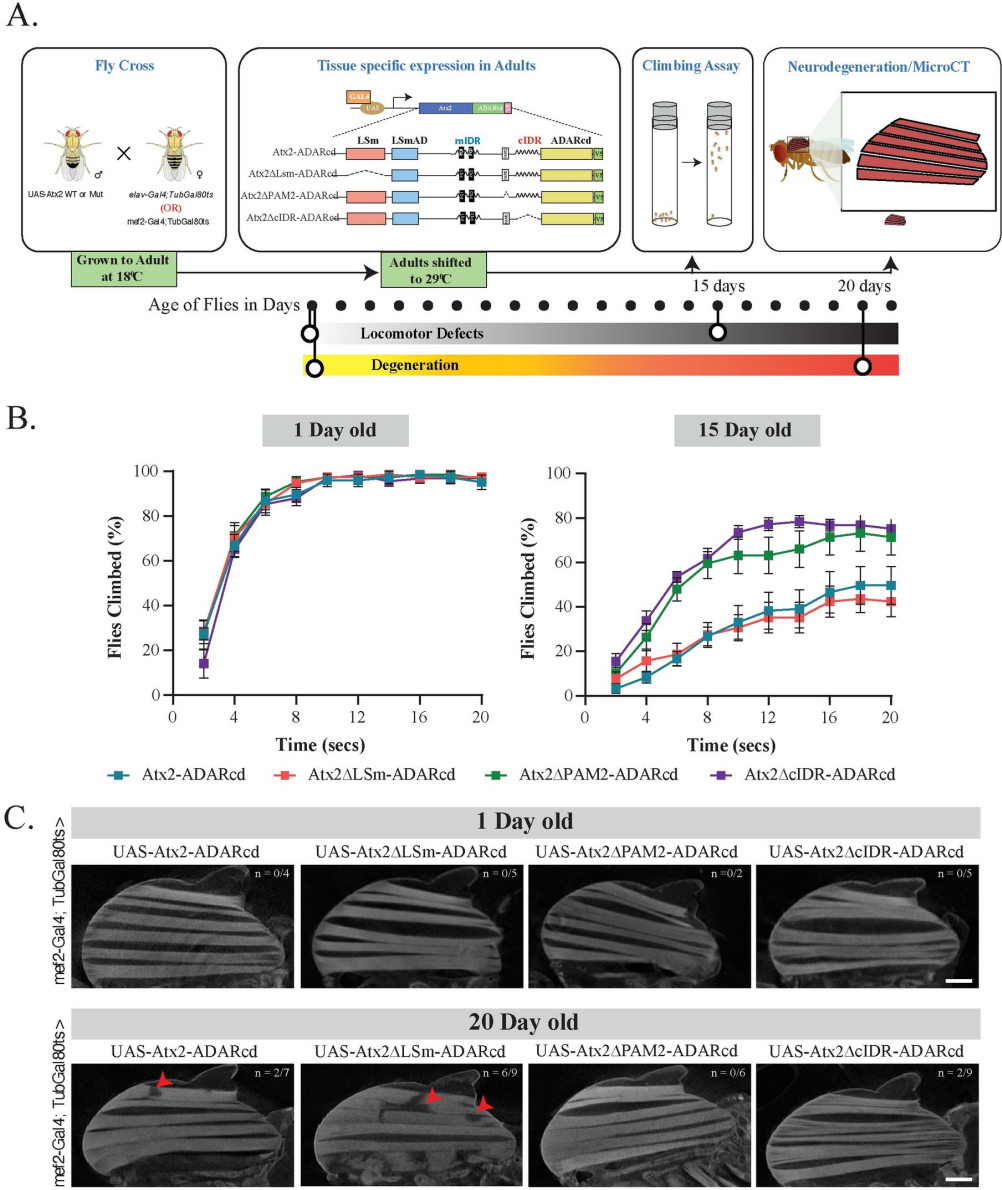
The IDR and PAM2 domains promote and the LSm domain inhibits neurodegeneration in *Drosophila*. (A) A schematic of the experimental design is shown. UAS-transgenes were crossed with *elav-Gal4; tub-Gal80*^*ts*^ or *mef2-Gal4*, *tub-Gal80*^*ts*^ and kept at 18°C till the adult flies emerged. The flies were shifted to 29°C for days shown with dots under the experimental design. Fly climbing or indirect flight muscle cytotoxicity was studied. (B) *Drosophila* climbing behavior assay was performed by driving UAS-transgene (Atx2WT, Atx2ΔcIDR, Atx2ΔPAM2 or Atx2ΔLSm) with *elav-Gal4*. A graph was plotted with number of flies (Y-axis) that crossed the 20ml mark at a given time (X-axis). (C) Cellular toxicity was measured by driving UAS-transgene (Atx2WT, Atx2ΔcIDR, Atx2ΔPAM2 or Atx2ΔLSm) with *mef2-Gal4*. Fly indirect flight muscles were imaged using micro-CT and the loss of muscle fibers is shown with solid red arrowheads. Micro-CT transverse views of the adult fly indirect flight muscles in the same assay are shown in VI Fig in S1 File. The notation n/n inserted in the figure represents the number of animals displaying a phenotype out of the total number of animals tested.

To examine how the different Atx2 domain deletions affect nervous system integrity and function over time, we combined a Gal4-responsive *UAS-Atx2* transgene with *elav-Gal4* and *TubGal80*^*ts*^. This allows us to use a temperature shift from 18°C to 29°C to induce *UAS-Atx2* transgene expression, specifically in the brains of adult flies (Fig 5A). We then analysed the rate at which flies climbed the walls of a glass cylinder, a surrogate measure of motor ability, one day and 15 days after transgene expression. All genotypes tested showed robust and comparable levels of climbing ability on day 1. Interesting variations were observed on day 15. The 15-day old flies expressing Atx2WT or Atx2ΔLSm showed a strong decline in climbing ability. In contrast, Atx2ΔcIDR flies showed a minimal decline (Fig 5B). These observations were in line with the effects of these Atx2 types on granule formation. Strikingly, flies expressing the Atx2ΔPAM2 variant, which formed compositionally distinct granules in S2 cells, showed no significant decline in climbing ability, suggesting that Atx2’s ability to promote progressive decline of neural function depends less on Atx2 granule formation and aggregation, and a bit more on its sequestration of critical translation factors such as PABP (and associated RNAs (Fig 5B). These observations support and extend prior work showing that heterologous overexpression of full-length, but not PAM2-domain deleted forms of mammalian ATXN2 enhances mammalian TDP-43-induced degeneration of the *Drosophila* compound eye [73]. They are also consistent with work in mice showing that PABPC1 sequestration in inclusions correlates strongly with the progression of neurodegeneration [94].

The conclusion, that PAM2-mediated interactions were required for progressive cytotoxicity, is further supported by a parallel series of experiments in which we used *mef2-Gal4* in place of *elav-Gal4*, to target *UAS-Atx2* transgene expression to *Drosophila* adult muscles (Fig 5C). Micro-computed tomography (micro-CT) scanning to visualize the integrity of flight muscle fibers in whole-mount preparations (see Methods) revealed degeneration of muscles expressing wild-type Atx2 in 20-day old flies. While there was more severe degeneration in Atx2ΔLSm expressing muscle, muscles similarly expressing Atx2ΔcIDR or Atx2ΔPAM2 forms showed no morphological defects (Fig 5C and VI Fig in S1 File).

## Discussion

The results described above provide three significant lines of insight. First, they support a model for sequential protein-protein interactions through which Ataxin-2 can regulate the translational states of mRNAs. Second, they show that the Ataxin-2 polypeptide contains distinct activities that promote or protect against neurodegeneration, pointing to the value of developing therapeutics that target specific Ataxin-2 interactions, beyond those that reduce overall levels of the protein. Third, the work identifies a novel molecular mechanism involving the PAM2 domain and PABP that contributes to the assembly of mRNP granules.

### Molecular mechanisms of Ataxin-2 function

Some RNA-binding proteins can remain associated with mRNAs across multiple stages: RNA processing, transport, translation, or translational control [2,95–99]. Ataxin-2 may be one such protein. It is a translational activator of the *Drosophila period* mRNA, a repressor of several miRNA reporters, a facilitator of neuronal mRNP-granule and stress-granule formation as well as a broad stabilizer of Ataxin-2 associated mRNAs [61,92,100–105]. While these different functions could represent different modes of engagement with distinct sets of mRNAs, the data are also consistent with another model. Sequential interactions mediated by different protein regions during mRNP modelling allow Ataxin-2 to contribute in multiple ways to translational control to a single mRNA.

Previous work has shown that Atx2 enhances *period* mRNA translation through a mechanism requiring LSm-domain interactions with a complex of LSM12 and TYF (Twenty Four) proteins associated with the 5’cap of the translating mRNA [100,105,106]. Given considerable supportive evidence for direct binding between the LSm domain and LSM12, we postulate that LSm-domain-LSM12 interactions occur in translating polysomes [84] and contribute to increased efficiency of translation.

This proposal is consistent with the observation that the LSm domain opposes the formation of mRNP granules, which usually contain translationally repressed mRNAs [83]. However, the LSm domain must also contribute to LSM12-independent functions, because LSm-domain deletions from *Drosophila* Atx2 cause lethality and LSM12 null mutants, while arrhythmic, are viable and fertile [106]. One possibility is that LSm domains additionally contribute, perhaps indirectly, to interactions with the DEAD-box helicase Me31B/DDX6 in a translational repressor complex [106,107]. Thus, we suggest that in the case of actively translating mRNAs, the Atx2 function is driven by LSm-domain association with LSM12 and translational initiators, and that LSM12 disengages from a translational initiation complex as the mRNA transitions into a repressed state driven by Me31B.

While polyA tails and PABP are known to support translation and the Ataxin-2 PAM2 domain is involved in targeting the protein to polysomes [84], existing data do not directly address how Ataxin-2 PAM2 motif interactions contribute to translational activation. One possibility, supported by observations on the *period* mRNA is that the PAM2 domain guides Ataxin-2 to the 3’UTR of its target mRNAs [100]. Our observation that PABP co-immunoprecipitates with mini-Ataxin2, show that Atx2PAM2:PABP interactions occur independently of and prior to mRNP granule formation. Recent findings that this association antagonizes the Ataxin-2 condensation [82] are consistent with a model in which the Atx2-PAM2 motif interacts with PABP in translating mRNAs to support efficient translation. However, in addition to supporting translation, PABP is also known to associate with translational repressors that could drive either mRNA deadenylation and/or storage [93,108,109]. We propose a dual role for Ataxin-2 associated with PABP in translational repression. First, when Ataxin-2 target mRNAs are not actively translated, then the mRNP through Me31B/DDX6 and PABP may recruit deadenylases to transition into either a translationally dormant or degradative state [106,109–111]. Second, Atx2-associated mRNA may move into mRNP granules whose formation is facilitated by Atx2 IDR-mediated condensation. We postulate that mRNAs in such assemblies are stored in a form that is protected from degradation. Although such a model aligns with our data, we recognize the absence of experimental evidence in the current study and acknowledge the necessity for extensive and rigorous testing within the life cycle of a single Ataxin-2 target mRNA.

### Implications for Ataxin-2 as a therapeutic target

Antisense Oligonucleotide (ASO) based therapeutic strategies that lower levels of ATXN2 are being developed for the treatment of ALS and SCA2 [72,77]. Our experiments provide a much finer grained analysis of the activities of Ataxin-2, suggesting that the function of the LSm domain should be spared, and that IDR-mediated assembly mechanisms and perhaps PAM2:PABP interactions should be most usefully targeted by therapeutics.

Our previous work showed that *atx2* mutants lacking the cIDR required for Ataxin-2 granule formation in *Drosophila* neurons and S2 cells, were resistant to neurodegeneration as assessed in *Drosophila* disease models [61,74]. We further showed that the LSm-domain antagonizes Ataxin-2 granule formation [83]. Here we advance the latter observation by demonstrating that Ataxin-2 forms lacking the LSm domain may more effectively cause cytotoxicity than the wild-type or IDR-deficient forms (Fig 5C). While Ataxin-2 is a largely disordered protein, structured regions within the protein may confer functionality by facilitating the formation of more stable complexes, thereby hindering aggregation. However, we remain uncertain whether the LSm domain alone can exert neuroprotective effects. These observations independently confirm our original conclusions and provide further support for a model in which the efficiency of mRNP assembly correlates with the speed and severity of neurodegenerative processes in *Drosophila*.

The importance of the PAM2 domain in promoting degeneration has been previously observed by experiments showing that heterologous expression of a pathogenic form of human Ataxin-2 lacking its PAM2 domain, but not the full-length form, suppresses cytotoxicity in *Drosophila* expressing human TDP-43 [73]. Our observations that the expression of Atx2ΔPAM2 is far less toxic than expression of wild-type Atx2 is consistent with this. The exogenous human ATXN2 assays however, did not examine the role of granule formation and contents for the phenotype and did not focus on the normal function and dysfunction of the native Atx2 in flies. Despite the high conservation of the structured domains of Ataxin-2 between human and *Drosophila*, our data shows that human ATXN2 does not form distinct granules in S2 cells and that this outcome is not dependent on the evolutionary divergence of the structured domains (panels A-B of V Fig in S1 File). Furthermore, the human gene is not sufficiently conserved to replace the essential functions of fly *atx2 in vivo* (panel C of V Fig in S1 File). Finally, by showing that Atx2ΔPAM2 forms compositionally different Ataxin-2 granules, our new data highlight the importance of specific granule components, and not granules *per se*, in neurodegenerative pathologies. Thus, while liquid-liquid transitions mediated by disordered domains could be a shared requirement for the formation of multiple types of mRNP granules, we speculate that each granule type, with distinctive composition, could preferentially support one or other type of proteinopathy [112,113].

### Structured interactions may determine mRNP granule composition

**Fig 6:**
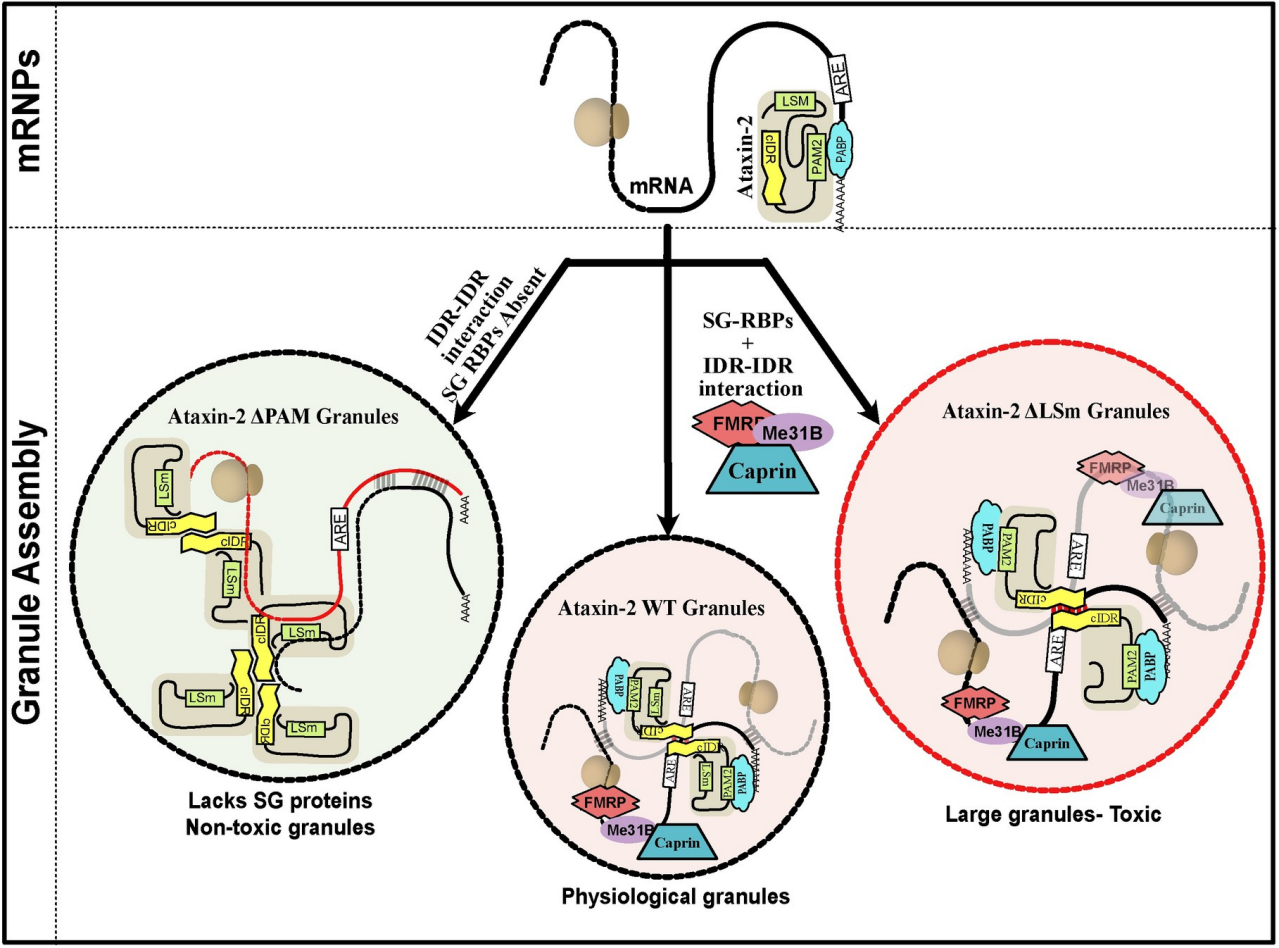
A model for the role of structured domains in Ataxin-2 determining its RNP composition. Ataxin-2 is recruited to mRNAs by RBPs during different stages of the mRNA life cycle. Ataxin-2 interacts with the 3’UTR of the subset of mRNA. Ataxin-2 requires the structured LSm and PAM2 domains for physiological granule assembly, where the cIDR along with RNA-RNA interaction stabilises the RNP condensation. Ataxin2-PAM2 domain determines the protein and RNA partners of the RNP granules. The PAM2 domain is essential for the recruitment of Ataxin-2 to SGs that also contain other RBPs (eg. Me31B, FMRP, Rox8, Rin and Caprin). The Ataxin-2ΔPAM2 granules which lacks several known SG proteins are non-toxic. While the LSm domain does not influence the composition of Ataxin-2 granules, Ataxin-2ΔLSm granules are larger and exhibit greater cytotoxicity.

Many lines of evidence argue that specific molecular interactions, e.g. mediated by structured domains of the P-body component Edc3 or the stress-granule components G3BP1 and Caprin, contribute to the mRNP granule formation [31,114]. In engineered systems, the condensation of RNA-binding proteins and mRNAs into granules has been clearly shown to depend on both traditional protein-protein interactions and on more promiscuous interactions between IDRs [115]. Our work now identifies the interactions between Ataxin-2’s PAM2 motif and PABP as a critical contributor to the assembly of Ataxin-2 containing mRNP granules. This suggests a mechanism by which the multiple interactions helps select mRNA and protein components of mRNP granules (Fig 6).

We suggest that Ataxin-2, guided by PAM2:PABP interactions and LSm domain interactions, recruits target mRNAs and associated proteins into translating mRNPs [84]. Under conditions where the translation is arrested, LSm-domain interactions are altered [106], and transcripts are released from stalled ribosomes. Base-pairing interactions between exposed mRNA, as well as interactions between Ataxin-2’s now accessible IDRs, contribute to the assembly of these mRNPs into granules. This logical sequence of events is consistent with: (a) TRIBE data showing a reduced number of edits of native Ataxin-2 target mRNAs by Atx2ΔPAM2-ADARcd; (b) the inability of mini-Atx2ΔPAM2 constructs to associate with SGs; and (c) the aberrant protein composition of granules induced by Atx2ΔPAM2 in S2 cells. The additional observation that Atx2ΔPAM2-ADARcd expression results in a large number of non-native mRNA edits, indicates that the PAM2:PABP interaction not only selects correct target mRNAs but also prevents Ataxin-2 engagement with incorrect mRNA target regions.

Our conclusion that Ataxin2-PAM2:PABP interactions are involved in the selection of mRNA components of RNP granules is superficially inconsistent with the observation that RNA components of native SGs can be predicted with remarkable accuracy on the basis of mRNA size. This argues for a primary role for RNA-RNA interactions in the SG assembly [7,8,116]. However, we note that experiments presented here do not address mechanisms by which mRNAs are selected into SGs. Instead, the TRIBE data address how Atx2-target mRNAs are selected into neuronal mRNP granules that exist in non-stressed cells *in vivo*, and microscopic studies analyse protein components of mRNP granules formed following Atx2 expression in S2 cells. Our experiments and observations therefore point to fundamental differences in mechanisms by which the assembly of neuronal granules, or granule types found in unstressed cells, may differ from those involved in stress-granule assembly. The regulation and composition of the former class could well rely extensively on specific protein-protein and protein-mRNA interactions, which may be revealed by future analyses of mechanisms by which such mRNP assemblies are formed *in vivo*.

## Materials and methods

### Cell culture, transfection and stress induction

*Drosophila* S2R+ cells were obtained from the DGRC, Indiana University, and were grown in Schneider’s S2 media with 10% FBS and 1% penicillin and streptomycin, at 25°C. Transfections were performed using either FugeneHD (Active Motif) or TransIT-X2 (Mirus) reagents at 2:1 ratio μl reagent to μg plasmid DNA for 24–72 h depending on downstream use. HEK293T cells from Adrian Bracken, Trinity College Dublin, were grown in Gibco Dulbecco’s Modified Eagle Media with 10% FBS, 2 mM l-glutamine addition, 1% penicillin and streptomycin, at 37°C and 5% CO_2_. U2OS cells from Martina Schroeder, Maynooth University, were grown at the same conditions as HEK293T. Mammalian cell transfections were carried out with 1 mM PEI (Polysciences) solution at 2:1 ratio μl reagent to μg plasmid DNA for 24-72h depending on the downstream use. For confocal imaging applications cells were grown in 24-well plates on glass cover-slips for 24 h before transfection for up to 48 h. For Western blotting and IP, cells were grown in 75 cm^2^ flasks until >80% confluent before transfection for up to 72 h before harvesting. Oxidative stress was induced in *Drosophila* S2R+ cells with addition of sodium arsenite solution to a final concentration of 500 μM in media for 3 h. In mammalian cells, oxidative stress was induced in the same way for 1 h.

### Western blotting and protein immunoprecipitation

Total protein extracts were prepared from S2 and HEK293 cells as described earlier [103]. Up to 10 μg total protein was loaded per well for detecting Ataxin-2-SNAP constructs, partner proteins and loading controls on 8–12% SDS-PAGE gels and transferred to nitrocellulose membranes. The blots were probed in 5% skim milk in PBS using rabbit anti-SNAP (1:1000), rabbit anti-PABP (1:1000), rabbit anti-LSM12 (1:1000) antibodies, and mouse anti-histone H3 (1:5000) and mouse anti-BAF155 (1:2000) loading control antibodies. Corresponding HRP-conjugated secondary antibodies were used at 1:10,000 dilution and developed using Pierce ECL western blotting substrate (ThermoFisher) as per the manufacturer’s instructions.

For Ataxin-2-SNAP immunoprecipitation, transfected cell lysates were normalised to the same volume and concentration, 10% of the volume was saved and diluted as an input control, and Chromotek anti-SNAP-tag conjugated agarose beads and IP kits were used according to the manufacturer’s specifications. Pulled-down proteins together with corresponding sample input controls were blotted as described above.

For fly brain protein western blotting, 20 freshly dissected fly brains per genotype were lysed in sample buffer through homogenisation and poaching. The mixture was spun down and supernatant was used for blotting as previously described with cell lysates.

### Immunohistochemistry and imaging of cultured cells

Transfected cells on coverslips were fixed with 4% paraformaldehyde in PBS solution for 15 min, followed by three 5 min washes in PBS. Permeabilization was performed on all cells with 0.5% TritonX100 in PBS solution for 3 min, before three more 5 min washes in PBS. Cells were blocked with 3% BSA in PBS for 1 h at room temperature before staining with primary antibodies at appropriate dilutions in 3% BSA overnight at 4°C. Corresponding fluorescent secondary antibodies in 3% BSA were used to stain the sample cells for 1 h at room temperature after primaries were washed off. Where SNAP-tagged proteins were being visualized, SNAPligand TMR-Star (NEB) or SNAP-surface-Alexa488 (NEB) were added at the secondary antibody staining stage. Following staining and washing, cells were mounted upside-down on microscopy slides in MOWIOL, allowed to cure for >12 h at 4°C, and imaged on a Zeiss LSM880 Airyscan/AiryscanFast confocal microscope with a 20x air objective.

### Bioimage analysis

Where relevant, Airyscan images were processed with Zen Black software (Zeiss) with recommended settings. Confocal microscopy images were analysed using macros within ImageJ/FIJI and Excel. Quantification of co-localisation was performed by comparing SG marker staining intensity profiles across a randomised selection of Ataxin-2 granules within transfected cells, with the intensity profile of the Ataxin-2 staining. Any signal 10% or higher than background (adjusted for fluorophore bleed through) was deemed evidence of colocalisation within that particular granule. For quantifying the exclusion of mini-Ataxin-2-SNAP constructs from stress induced granules the Caprin or G3BP1 staining was used as independent identifier of SGs and Ataxin-2 profiles were compared to them. 48–120 granules were quantified in each co-staining (Fig 2), and 28–70 granules were quantified for each construct transfection (Fig 4).

### Crystal structure threading

Threading of the *Drosophila* PAM2 peptide bound to the MLLE domain of PABPC1 was performed using the Swiss-PdbViewer software, based on the human crystal structure of the complex obtained from PDB, identified as 3KTR [90].

### Experimental fly crosses

*Drosophila* stocks were maintained at 25°C in corn meal agar. Strains homozygous for UAS transgenes were crossed with *elav-Gal4* and *tub-Gal80ts* at 18°C till the adult fly emerged. The flies were shifted to 29°C for 5 days before processing for RNA extraction for TRIBE experiments. The climbing behaviour experiments were performed on flies kept at 29°C for either 1 or 15 days. For microCT experiments, the UAS-transgenes were crossed with *mef2Gal4* and *tub-Gal80ts* at 18°C and the adult flies were transferred to 29°C for 1 day or 20 days.

### Generation of *atx2* locus replacement flies

*Drosophila atx2* genetic locus replacement flies were generated using two rounds of *in vivo* genome editing. First, CRISPR/Cas9 technique was utilised to heterozygously replace the open reading frame of *atx2* with an attP>3x P3 dsRED<attP cassette to generate a null allele insertion site with a selectable marker. Balanced insertion cassette fly eggs were then injected in the pole with PhiC31B integrase and plasmids with SNAP-tagged Ataxin-2 forms flanked by inverted attB sites to induce replacement of the cassette. Flies were screened for loss of dsRED expression in eyes, and by PCR and sequencing for the correct orientation and sequence of integrated fly *atx2* cDNA, human ATXN2 cDNA, and fly mini-*atx2* within the *atx2* locus.

### RNA extraction from brain and NGS

Around 10–12 adult brains were dissected in RNA Later for total RNA isolation. RNA was isolated using TRIzol reagent (Invitrogen) as per the manufacturer’s protocol. Poly(A)enriched mRNA was used to prepare Illumina libraries using the NEBNext Ultra II Directional RNA Library Prep kit (E7765L). Atx2ΔPAM2-ADARcd and samples were sequenced with Illumina HiSeq PE Rapid Cluster Kit v2 (PE-402-4002) to generate 2×100 paired-end strand-specific data using the Illumina HiSeq 2500 sequencing platform.

### TRIBE data analysis

The sequencing reads obtained had a mean quality score (Q-Score) > = 37. Analysis of the TRIBE data was performed as described previously [83,87]. Briefly, the reference genome and gtf file of *Drosophila melanogaster*, version dm6, were downloaded from the UCSC genome browser. Raw sequencing reads were mapped using TopHat2 [117] with the parameters ‘—library-type fr-firststrand -m 1 N 3 -read-edit-dist 3 p 5 g 2 -I 50000—microexon-search—no-coverage-search -G dm6_genes.gtf’. Only uniquely mapped reads are considered for editing analysis. A table of raw and mapped reads is included in Table A in S1 File. A threshold file was created by ensuring only edits with coverage of at least 20 reads and 15% edits were retained. All the TRIBE experiments were performed in duplicates, and only the edits identified in both replicates above the edit threshold are reported.

### Climbing assay

Appropriately aged adult *Drosophila* was transferred to a 50 ml graduated glass measuring cylinder for the climbing assay and sealed with a cotton plug. A digital video camera was positioned to record the vials. The assay was initiated by tapping the cylinder against a foam pad to collect the flies to the bottom of the cylinder and the flies were allowed to climb the cylinder with video being recorded for ~30 s. The number of flies that crossed the 20 ml mark (~5.5cm) was counted over time and the data was plotted against the time using GraphPad prism. Average of 3 trials were used for each biological replicate. 7–10 biological replicates were used for each genotype.

### Sample preparation and scanning for microCT

*Drosophila* indirect flight muscle microCT was carried out as described previously [118]. Briefly, animals were anesthetized on ice and fixed in PBS containing 4% paraformaldehyde (PFA). Thoraces were dissected and stained using 1% elemental iodine (1.93900.0121, Emparta, Merck) with 2% potassium iodide (no. 15 724, Qualigens) dissolved in PBS. The stained samples were washed in PBS and embedded in petroleum jelly. MicroCT scanning was carried out at 40 kV, 250 μA, on Bruker Skyscan-1272.

## Supporting information

S1 File I Fig: Additional data relating to fly brain TRIBE experiments shown in Fig 1. (A) Western Blots showing the comparable expression levels of Atx2-ADARcd forms in *Drosophila* brains at expression permissive temperature. WT and Atx2ΔPAM2 -ADARcd are both not expressed in brains in flies raised for 5 days post-ecclosure at 18°C due to Gal80ts inactivation of elav-Gal4. In flies raised at the permissive temperature of 30°C there are highly similar expression levels of the two forms of V5 tagged Atx2 in normalised samples (loading control: Actin). Differences in the editing efficiency and targeting of Atx2 forms are not tied to differential expression of the transgenes. (B) Expression level normalization using FPKM (Fragments Per Kilobase of transcript per Million mapped reads) shows equal expression of Atx2WT -ADARcd and Atx2ΔPAM2 -ADARcd. (C) Correlation analyses between biological replicates and across genotypes for TRIBE experiments. These analyses reveal much stronger correlation between the replicates of the same genotype than across different genotypes (Atx2WT-ADARcd and Atx2ΔPAM2-ADARcd). (D) Scatter plot comparing mRNA expression levels of Atx2ΔPAM2 TRIBE targets with expression levels of all sequenced mRNAs. Atx2ΔPAM2 targets are distributed across the expression spectrum suggesting it is sensitive and specific to the targets and not biased towards highly expressed genes. R1 and R2 are biological replicates for Atx2ΔPAM2 TRIBE. **II Fig:** Co-localisation quantification for Figs 2 and 4. (A) Normalised profile plots of Atx2-GFP granules in S2 cells as shown in Fig 2. Within representative granules of wild type Atx2-GFP (green line), SG components Caprin, dFMRP, PABP, Me31B, and Rox8 show largely overlapping enrichment of fluorescence profile along a line bisecting a granule after immunohistochemistry and imaging (purple line). In Atx2ΔPAM2-GFP granules, this colocalization of fluorescence signals is not seen in the case of Caprin, dFMRP and PABP, suggesting these components are not enriched in these granules above background level. (B) Quantification of co-localization for Fig 2. N = 48–120 images of Atx2-GFP granules were randomly selected for each co-staining and analysed for signal co-enrichment (see methods) in the case of each component assayed. (C) Quantification of Atx2 construct inclusion in SGs for Fig 4. N = 28–70 images of SGs in arsenite stressed S2 cells (marked by anti-Caprin staining) and U2OS cells (marked by anti-G3BP1 staining) were randomly selected for each Ataxin-2 construct assayed and were analysed for Mini Ataxin-2-SNAP allele signal co-enrichment (see methods). **III Fig:** Atx2ΔLSm granules in S2 cells do not show significantly altered protein contents compared to wild-type Atx2, as shown in Fig 2. (A) Caprin, dFMRP, PABP, Me31B, and Rox8 colocalize with overexpressed Atx2ΔLSm GFP, suggesting that the granules formed contain a similar set of components as Atx2 granules. (B) Quantification of co-localization with Atx2ΔLSm granules from randomly selected cells and analysed for signal co-enrichment. (C) Arsenite-stressed S2 cells were randomly selected and Atx2ΔLSm granules were assayed for signal co-localisation with stress granule components (n = 24 for each protein). Percentages of granules where signal enrichment coincided were calculated. It should be noted that Atx2 granules do not sequester the majority of the endogenous components stained for, leading to a high, diffuse background staining. **IV Fig:** Additional controls, data and replicates relating to Figs 2 and 3. (A) Overexpression of Atx2 mutants in S2R+ cells does not affect the endogenous expression of Atx2 interactors and SG proteins. Thus observations from transfected S2R+ cells in Figs 2 and 3 are likely not a result of disrupted partner or in general protein expression. (B) Biological replicate of Fig 3C. (C-D) Replicates of S2 cell IP-WB of human analogous *Drosophila* mini-Atx2 PAM2 point mutant constructs. These point mutants disrupt the binding between fly Atx2 and PABP. (E-F) The ATXN2 PAM2 and the PABPC1 MLLE domain are highly conserved from fly to human. (E) The ATXN2 PAM2 domain exhibits high sequence similarity where the key MLLE domain hydrophobic binding residues leucine 914 and phenylalanine 921 (human ATXN2 numbering) are conserved from *Drosophila* to humans. (F) Its binding partner, the PABPC1 MLLE domain, is also highly conserved from *Drosophila* to human. Sequence IDs: Q8SWR8 (Atx2_DROME), Q99700 (ATXN2_HUMAN), Q8WWM7 (ATX2L_HUMAN), P21187 (PABP_DROME), P11940 (PABP1_HUMAN). **V Fig:** Human ATXN2 and *Drosophil*a Atx2 exhibits high structured domain homology however are not functionally interchangeable in cell assays and in flies. (A) Table of Clustal Ω amino acid similarity percentage between the three human and *Drosophila* Ataxin-2 structured domains. (B) S2 cell expression assays showing the granule forming phenotype of full length fly Atx2 is not conserved with human full length ATXN2. Structured domain conservation is not sufficient to replicate the fly Atx2 phenotype while domain swapped chimeric protein (human ATXN2 cDNA with swapped out structured domains to fly Atx2 sequences) also fails to form distinct granules. Scale bar = 5μm. (C) Summary of the strategy and results of atx2 gene ORF replacement assay in *Drosophil*a. The *atx2* locus was edited with CRISPR/Cas9 to exchange the ORF with a site-directed integration and marker cassette, generating an atx2 null allele. This cassette was subsequently swapped out with either fly Atx2 cDNA, human ATXN2 cDNA or fly mini-Atx2 sequences using site-directed integration. The full-length Atx2 cDNA allele from *Drosophila* supported the survival of animals when present in the homozygous state. However, neither the truncated *Drosophila* mini-Atx2 nor the full-length human ATXN2 cDNA were able to rescue survival in homozygous states. **VI Fig:** Transverse view of *Drosophila* indirect flight muscle imaged using micro-CT shows cellular toxicity. (A) As in Fig 6C, driving UAS-transgene (Atx2WT, Atx2ΔcIDR, Atx2ΔPAM2 or Atx2ΔLSm) with *mef2-Gal4* show normal muscles on day 1. (B) Expression of wild-type and Atx2ΔLSm transgene for 20 days show loss of muscle fibers, indicated with solid red arrowheads. Expression of Atx2ΔPAM2 and Atx2ΔcIDR for 20 days show no visible phenotype. The notation n/n inserted in the figure represents the number of animals displaying a phenotype out of the total number of animals tested. **Table A.** NGS Sequencing read numbers and quality. **Table B.** The targets common between Atx2 wild-type and ΔPAM2 are shown in bold text.
(DOCX)

S2 File Contains information on reagents used for the study. (DOCX)

S1 Dataset The file contains the raw data used for the graphs presented in Figs 1 and 5. (ZIP)
