# Supplementary material for: Structured and disordered regions of Ataxin-2 contribute differently to the specificity and efficiency of mRNP granule formation
Source: PLoS Genet. 2024 May 20;20(5):e1011251. doi: 10.1371/journal.pgen.1011251 (PMC11166328; doi:10.1371/journal.pgen.1011251)
Supplement: S1 File — (A) Western Blots showing the comparable expression levels of Atx2-ADARcd forms in Drosophila brains at expression permissive temperature. WT and Atx2ΔPAM2 -ADARcd are both not expressed in brains in flies raised for 5 days post-ecclosure at 18°C due to Gal80ts inactivation of elav-Gal4. In flies raised at the permissive temperature of 30°C there are highly similar expression levels of the two forms of V5 tagged Atx2 in normalised samples (loading control: Actin). Differences in the editing efficiency and targeting of Atx2 forms are not tied to differential expression of the transgenes. (B) Expression level normalization using FPKM (Fragments Per Kilobase of transcript per Million mapped reads) shows equal expression of Atx2WT -ADARcd and Atx2ΔPAM2 -ADARcd. (C) Correlation analyses between biological replicates and across genotypes for TRIBE experiments. These analyses reveal much stronger correlation between the replicates of the same genotype than across different genotypes (Atx2WT-ADARcd and Atx2ΔPAM2-ADARcd). (D) Scatter plot comparing mRNA expression levels of Atx2ΔPAM2 TRIBE targets with expression levels of all sequenced mRNAs. Atx2ΔPAM2 targets are distributed across the expression spectrum suggesting it is sensitive and specific to the targets and not biased towards highly expressed genes. R1 and R2 are biological replicates for Atx2ΔPAM2 TRIBE. II Fig: Co-localisation quantification for Figs 2 and 4. (A) Normalised profile plots of Atx2-GFP granules in S2 cells as shown in Fig 2. Within representative granules of wild type Atx2-GFP (green line), SG components Caprin, dFMRP, PABP, Me31B, and Rox8 show largely overlapping enrichment of fluorescence profile along a line bisecting a granule after immunohistochemistry and imaging (purple line). In Atx2ΔPAM2-GFP granules, this colocalization of fluorescence signals is not seen in the case of Caprin, dFMRP and PABP, suggesting these components are not enriched in these granules above background level. (B) Quantif [file pgen.1011251.s001.docx]

**SUPPLEMENTARY FIGURES:**

**I_Fig.tif**

**
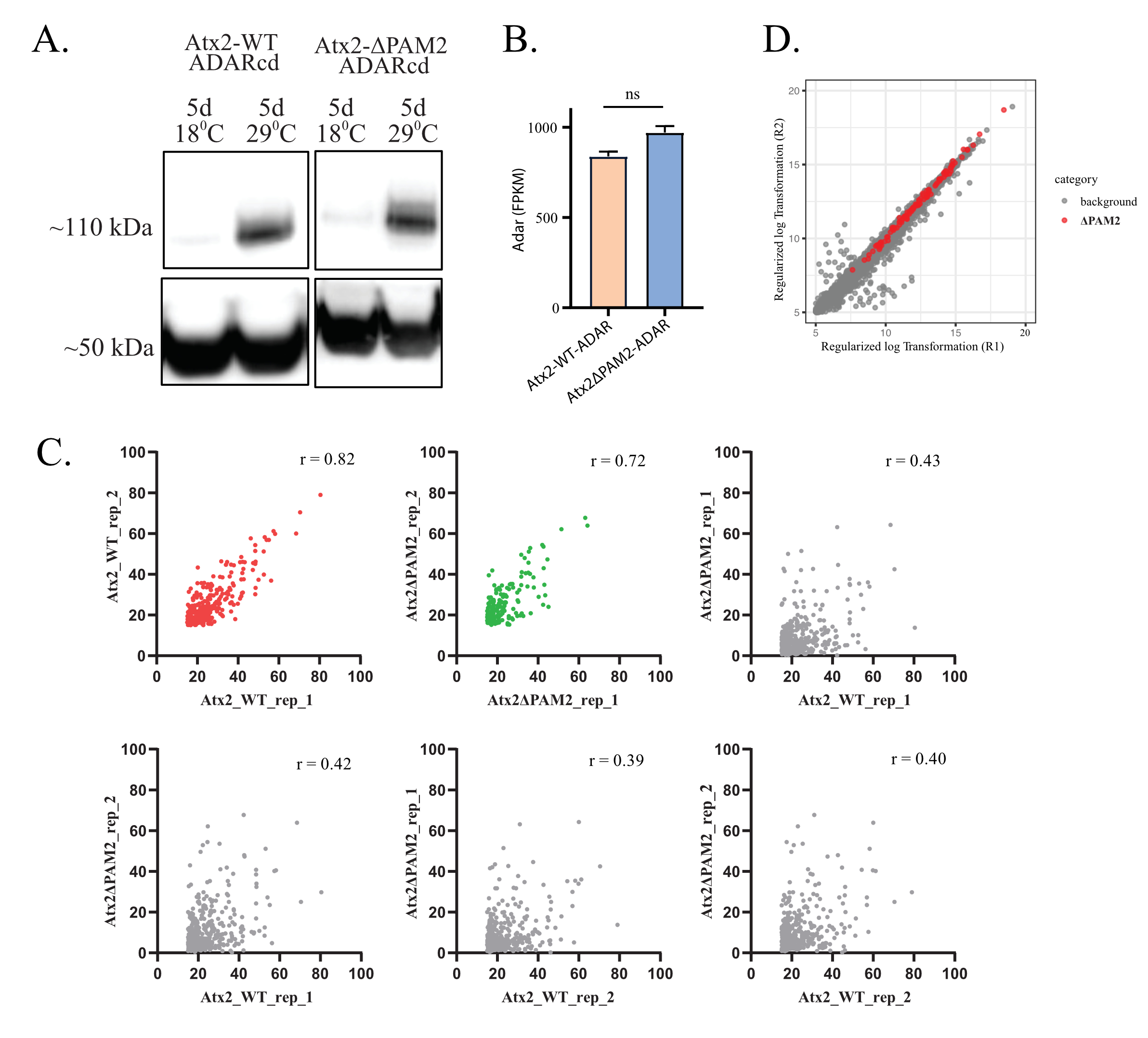
**

**I Fig:**  Additional data relating to fly brain TRIBE experiments shown in Fig 1. (A) Western Blots showing the comparable expression levels of Atx2-ADARcd forms in *Drosophila* brains at expression permissive temperature. WT and ΔPAM2 Atx2-ADARcd are both not expressed in brains in flies raised for 5 days post-ecclosure at 18°C due to Gal80ts inactivation of elav-Gal4. In flies raised at the permissive temperature of 30°C there are highly similar expression levels of the two forms of V5 tagged Atx2 in normalised samples (loading control: Actin). Differences in the editing efficiency and targeting of Atx2 forms are not tied to differential expression of the transgenes. (B) Expression level normalization using FPKM (Fragments Per Kilobase of transcript per Million mapped reads) shows equal expression of WT-Atx2-ADARcd and ΔPAM2-Atx2-ADARcd. (C) Correlation analyses between biological replicates and across genotypes for TRIBE experiments. These analyses reveal much stronger correlation between the replicates of the same genotype than across different genotypes (Atx2-WT-ADARcd and Atx2ΔPAM2-ADARcd). (D) Scatter plot comparing mRNA expression levels of Atx2ΔPAM TRIBE targets with expression levels of all sequenced mRNAs. Atx2ΔPAM2 targets are distributed across the expression spectrum suggesting it is sensitive and specific to the targets and not biased towards highly expressed genes. R1 and R2 are biological replicates for Atx2ΔPAM TRIBE.

**II_Fig.tif**


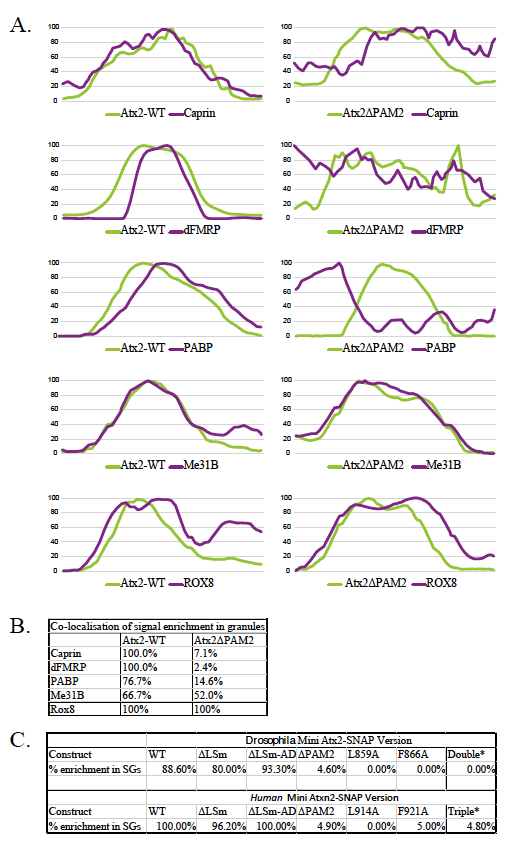


**II Fig:** Co-localisation quantification for Figs 2 and 4. (A) Normalised profile plots of Atx2-GFP granules in S2 cells as shown in Fig 2. Within representative granules of wild type Atx2-GFP (green line), SG components Caprin, dFMRP, PABP, Me31B, and Rox8 show largely overlapping enrichment of fluorescence profile along a line bisecting a granule after immunohistochemistry and imaging (purple line). In Atx2ΔPAM2-GFP granules, this colocalization of fluorescence signals is not seen in the case of Caprin, dFMRP and PABP, suggesting these components are not enriched in these granules above background level. (B) Quantification of co-localization for Fig 2. N = 48-120 images of Atx2-GFP granules were randomly selected for each co-staining and analysed for signal co-enrichment (see methods) in the case of each component assayed. (C) Quantification of Atx2 construct inclusion in SGs for Fig 4. N = 28-70 images of SGs in arsenite stressed S2 cells (marked by anti-Caprin staining) and U2OS cells (marked by anti-G3BP1 staining) were randomly selected for each Atx2 construct assayed and were analysed for Mini Atx2-SNAP allele signal co-enrichment (see methods).

**III_Fig.tif**


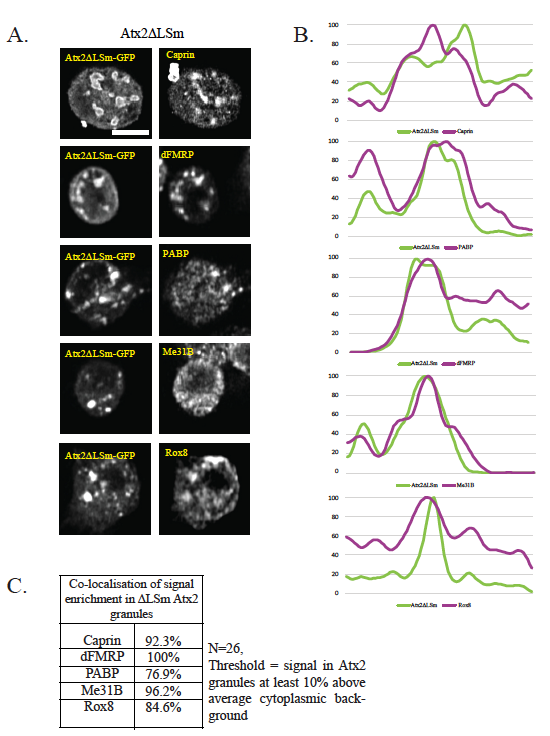


**III Fig:** Atx2ΔLSm granules in S2 cells do not show significantly altered protein contents compared to wild-type Atx2, as shown in Fig 2. (A) Caprin, dFMRP, PABP, Me31B, and Rox8 colocalize with overexpressed Atx2ΔLSm GFP, suggesting that the granules formed contain a similar set of components as Atx2 granules. (B) Quantification of co-localization with Atx2ΔLSm granules from randomly selected cells and analysed for signal co-enrichment. (C) Arsenite-stressed S2 cells were randomly selected and Atx2ΔLSm granules were assayed for signal co-localisation with stress granule components (n=24 for each protein). Percentages of granules where signal enrichment coincided were calculated. It should be noted that Atx2 granules do not sequester the majority of the endogenous components stained for, leading to a high, diffuse background staining.

**IV_Fig.tif**


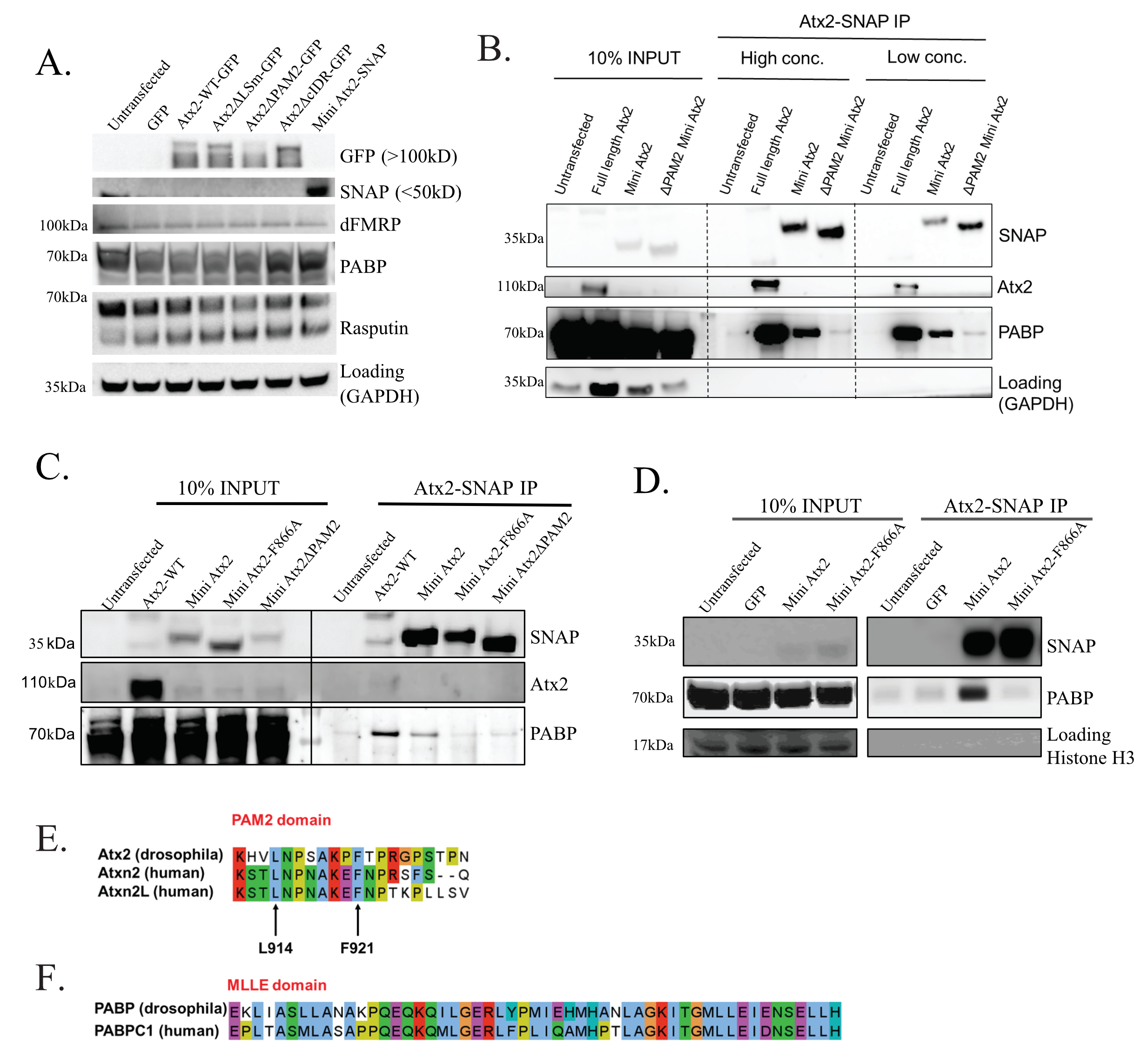


**IV Fig:** Additional controls, data and replicates relating to Figs 2 and 3. (A) Overexpression of Atx2 mutants in S2R+ cells does not affect the endogenous expression of Atx2 interactors and SG proteins. Thus observations from transfected S2R+ cells in Figs 2 and 3 are likely not a result of disrupted partner or in general protein expression. (B) Biological replicate of Figure 3C. (C-D) Replicates of S2 cell IP-WB of human analogous Drosophila mini-Atx2 PAM2 point mutant constructs. These point mutants disrupt the binding between fly Atx2 and PABP. (E-F) The ATXN2 PAM2 and the PABPC1 MLLE domain are highly conserved from fly to human. (E) The ATXN2 PAM2 domain exhibits high sequence similarity where the key MLLE domain hydrophobic binding residues leucine 914 and phenylalanine 921 (human ATXN2 numbering) are conserved from Drosophila to humans. (F) Its binding partner, the PABPC1 MLLE domain, is also highly conserved from Drosophila to human. Sequence IDs: Q8SWR8 (Atx2_DROME), Q99700 (ATXN2_HUMAN), Q8WWM7 (ATX2L_HUMAN), P21187 (PABP_DROME), P11940 (PABP1_HUMAN).

**V_Fig.tif**


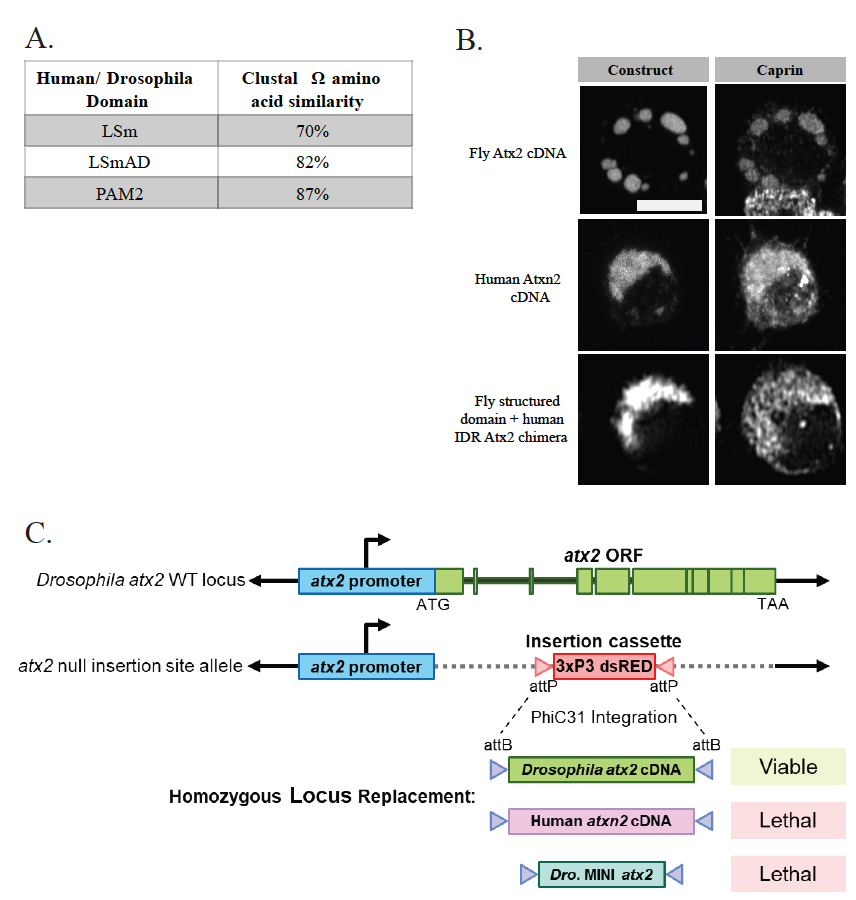


**V Fig:** Human ATXN2 and *Drosophil*a Atx2 exhibits high structured domain homology however are not functionally interchangeable in cell assays and in flies. (A) Table of Clustal Ω amino acid similarity percentage between the three human and *Drosophila* Ataxin-2 structured domains. (B) S2 cell expression assays showing the granule forming phenotype of full length fly Atx2 is not conserved with human full length ATXN2. Structured domain conservation is not sufficient to replicate the fly Atx2 phenotype while domain swapped chimeric protein (human ATXN2 cDNA with swapped out structured domains to fly Atx2 sequences) also fails to form distinct granules. Scale bar = 5μm. (C) Summary of the strategy and results of atx2 gene ORF replacement assay in *Drosophil*a. The atx2 locus was edited with CRISPR/Cas9 to exchange the ORF with a site-directed integration and marker cassette, generating an atx2 null allele. This cassette was subsequently swapped out with either fly Atx2 cDNA, human ATXN2 cDNA or fly mini-Atx2 sequences using site-directed integration. The full-length Atx2 cDNA allele from *Drosophila* supported the survival of animals when present in the homozygous state. However, neither the truncated *Drosophila* mini-Atx2 nor the full-length human Atxn2 cDNA were able to rescue survival in homozygous states.

**VI_Fig.tif**


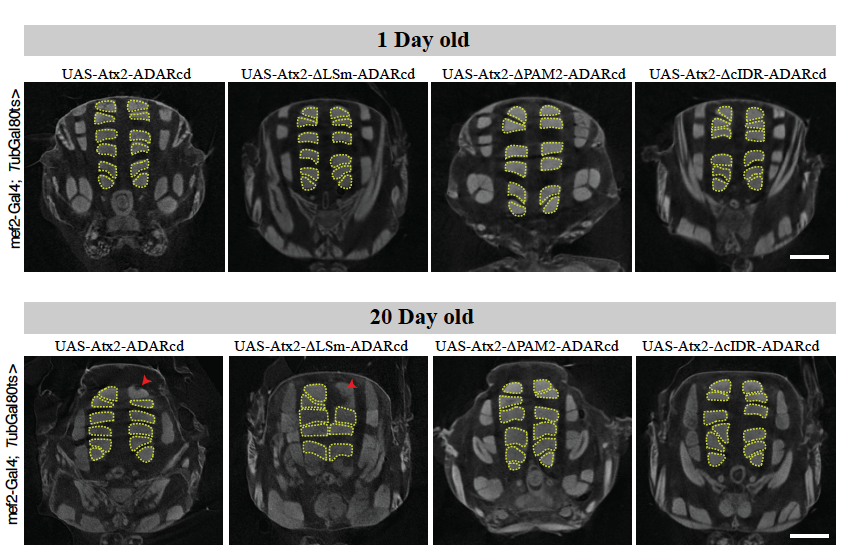


**VI Fig:** Transverse view of *Drosophila* indirect flight muscle imaged using micro-CT shows cellular toxicity. (A) As in Fig 6C, driving UAS-transgene (Atx2WT, Atx2ΔcIDR, Atx2ΔPAM or Atx2ΔLSm) with *mef2-Gal4* show normal muscles on day 1. (B) Expression of wild-type and Atx2ΔLSm transgene for 20 days show loss of muscle fibers, indicated with solid red arrowheads. Expression of Atx2ΔPAM2 and Atx2ΔcIDR for 20 days show no visible phenotype. The notation n/n inserted in the figure represents the number of animals displaying a phenotype out of the total number of animals tested.

**Table A:** NGS Sequencing read numbers and quality.


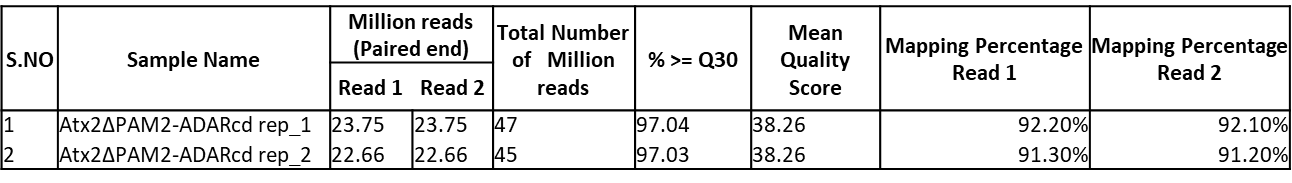


**Table B:** The targets common between Atx2 wild-type and del-PAM2 are shown in bold text.

| **Chr** | **Start** | **End** | **Genes** | **Replicate 1 edit percentage** | **Replicate 2 edit percentage** | **Average edit percentage** | **Chr_coordinate** |
| --- | --- | --- | --- | --- | --- | --- | --- |
| **chr2L** | **1009080** | **1009081** | **IA-2** | **17.1** | **15.5** | **16.3** | **chr2L_1009081_IA-2** |
| **chr2L** | **1009440** | **1009441** | **IA-2** | **31.7** | **38.8** | **35.25** | **chr2L_1009441_IA-2** |
| **chr2L** | **1011482** | **1011483** | **IA-2** | **31.7** | **34.7** | **33.2** | **chr2L_1011483_IA-2** |
| chr2L | 13505104 | 13505105 | B4 | 22.4 | 17.48 | 19.94 | chr2L_13505105_B4 |
| **chr2L** | **17188307** | **17188308** | **beat-IIIc** | **18** | **21.7** | **19.85** | **chr2L_17188308_beat-IIIc** |
| chr2L | 19746161 | 19746162 | CG10631 | 25.65 | 19.4 | 22.52 | chr2L_19746162_CG10631 |
| **chr2L** | **20056018** | **20056019** | **sNPF** | **16** | **24.3** | **20.15** | **chr2L_20056019_sNPF** |
| chr2L | 7891375 | 7891376 | Snoo | 18.5 | 16.7 | 17.6 | chr2L_7891376_Snoo |
| chr2L | 8113160 | 8113161 | Bsg | 17.4 | 19.05 | 18.23 | chr2L_8113161_Bsg |
| **chr2L** | **8113894** | **8113895** | **Bsg** | **16.82** | **28.7** | **22.76** | **chr2L_8113895_Bsg** |
| chr2L | 8116005 | 8116006 | Bsg | 18.2 | 27.4 | 22.8 | chr2L_8116006_Bsg |
| chr2L | 9256901 | 9256902 | Ggamma30A | 19.5 | 32.6 | 26.05 | chr2L_9256902_Ggamma30A |
| **chr2L** | **9292530** | **9292531** | **Ggamma30A** | **42** | **54.4** | **48.2** | **chr2L_9292531_Ggamma30A** |
| **chr2L** | **9292829** | **9292830** | **Ggamma30A** | **42.6** | **53.55** | **48.08** | **chr2L_9292830_Ggamma30A** |
| chr2L | 9295031 | 9295032 | Ggamma30A | 23.5 | 26 | 24.75 | chr2L_9295032_Ggamma30A |
| chr2R | 12085463 | 12085464 | jeb | 16 | 28.4 | 22.2 | chr2R_12085464_jeb |
| **chr2R** | **13513577** | **13513578** | **Vmat** | **64.2** | **63.9** | **64.05** | **chr2R_13513578_Vmat** |
| chr2R | 13513654 | 13513655 | Vmat | 27.7 | 33.45 | 30.58 | chr2R_13513655_Vmat |
| **chr2R** | **13513732** | **13513733** | **Vmat** | **24** | **29.75** | **26.88** | **chr2R_13513733_Vmat** |
| **chr2R** | **13513762** | **13513763** | **Vmat** | **44.55** | **47.3** | **45.92** | **chr2R_13513763_Vmat** |
| **chr2R** | **13514350** | **13514351** | **Vmat** | **37.7** | **38.5** | **38.1** | **chr2R_13514351_Vmat** |
| chr2R | 13519080 | 13519081 | Vmat | 18.1 | 20.8 | 19.45 | chr2R_13519081_Vmat |
| **chr2R** | **23690701** | **23690702** | **Pal2** | **17.3** | **17.2** | **17.25** | **chr2R_23690702_Pal2** |
| chr2R | 24213943 | 24213944 | CG30419 | 27.8 | 22.98 | 25.39 | chr2R_24213944_CG30419 |
| chr2R | 24214234 | 24214235 | CG30419 | 24.2 | 26.1 | 25.15 | chr2R_24214235_CG30419 |
| chr2R | 24214648 | 24214649 | CG30419 | 16.4 | 15.9 | 16.15 | chr2R_24214649_CG30419 |
| chr2R | 24215193 | 24215194 | CG30419 | 43.5 | 29.7 | 36.6 | chr2R_24215194_CG30419 |
| chr2R | 24229341 | 24229342 | CG30419 | 20.8 | 16.3 | 18.55 | chr2R_24229342_CG30419 |
| chr2R | 6914804 | 6914805 | CG30158 | 16.4 | 16 | 16.2 | chr2R_6914805_CG30158 |
| **chr2R** | **6920815** | **6920816** | **CG30158** | **33.92** | **40.48** | **37.2** | **chr2R_6920816_CG30158** |
| **chr2R** | **6921435** | **6921436** | **CG30158** | **17.5** | **41.9** | **29.7** | **chr2R_6921436_CG30158** |
| **chr2R** | **6921976** | **6921977** | **CG30158** | **36** | **40.2** | **38.1** | **chr2R_6921977_CG30158** |
| **chr2R** | **7718070** | **7718071** | **CG18812** | **15.9** | **19.7** | **17.8** | **chr2R_7718071_CG18812** |
| chr2R | 9473772 | 9473773 | Camta | 21.75 | 30.18 | 25.96 | chr2R_9473773_Camta |
| chr2R | 9479421 | 9479422 | Camta | 23.1 | 26 | 24.55 | chr2R_9479422_Camta |
| **chr2R** | **9480019** | **9480020** | **Camta** | **32** | **41** | **36.5** | **chr2R_9480020_Camta** |
| **chr2R** | **9910328** | **9910329** | **FMRFa** | **42.5** | **25** | **33.75** | **chr2R_9910329_FMRFa** |
| **chr3L** | **11498209** | **11498210** | **chrb** | **15.82** | **20.5** | **18.16** | **chr3L_11498210_chrb** |
| chr3L | 12267895 | 12267896 | CG32100 | 20 | 16.1 | 18.05 | chr3L_12267896_CG32100 |
| chr3L | 1504295 | 1504296 | Psa | 18.6 | 15.9 | 17.25 | chr3L_1504296_Psa |
| chr3L | 1521650 | 1521651 | Psa | 18.5 | 34.6 | 26.55 | chr3L_1521651_Psa |
| chr3L | 1543675 | 1543676 | CG7852 | 15.5 | 22 | 18.75 | chr3L_1543676_CG7852 |
| chr3L | 17062355 | 17062356 | Rbp6 | 15.2 | 18.5 | 16.85 | chr3L_17062356_Rbp6 |
| chr3L | 17147382 | 17147383 | Rbp6 | 18.23 | 26.62 | 22.42 | chr3L_17147383_Rbp6 |
| **chr3L** | **17345219** | **17345220** | **Mip** | **29.9** | **27.2** | **28.55** | **chr3L_17345220_Mip** |
| **chr3L** | **17345290** | **17345291** | **Mip** | **17.9** | **16.9** | **17.4** | **chr3L_17345291_Mip** |
| **chr3L** | **19066983** | **19066984** | **Mkp3** | **18.9** | **15.7** | **17.3** | **chr3L_19066984_Mkp3** |
| chr3L | 21494821 | 21494822 | Hr78 | 27.1 | 18.4 | 22.75 | chr3L_21494822_Hr78 |
| chr3L | 21831417 | 21831418 | CG7148 | 15.8 | 28.6 | 22.2 | chr3L_21831418_CG7148 |
| chr3L | 21930851 | 21930852 | mub | 15 | 17.2 | 16.1 | chr3L_21930852_mub |
| chr3L | 21931110 | 21931111 | mub | 15.1 | 20.1 | 17.6 | chr3L_21931111_mub |
| chr3L | 22061206 | 22061207 | Oct-TyrR | 15.4 | 16.7 | 16.05 | chr3L_22061207_Oct-TyrR |
| chr3L | 22877661 | 22877662 | Chro | 35.3 | 45.7 | 40.5 | chr3L_22877662_Chro |
| chr3L | 23148124 | 23148125 | CG32350 | 27.4 | 35.3 | 31.35 | chr3L_23148125_CG32350 |
| chr3L | 23747549 | 23747550 | CG17698 | 40.27 | 28.95 | 34.61 | chr3L_23747550_CG17698 |
| chr3L | 23934990 | 23934991 | CG40470 | 23.5 | 22.05 | 22.77 | chr3L_23934991_CG40470 |
| chr3L | 3910071 | 3910072 | Eip63F-1 | 20 | 24.5 | 22.25 | chr3L_3910072_Eip63F-1 |
| **chr3L** | **3954338** | **3954339** | **CG12605** | **35.2** | **40.8** | **38** | **chr3L_3954339_CG12605** |
| **chr3L** | **3954933** | **3954934** | **CG12605** | **35.42** | **51.1** | **43.26** | **chr3L_3954934_CG12605** |
| chr3L | 3957068 | 3957069 | CG12605 | 21.8 | 26.7 | 24.25 | chr3L_3957069_CG12605 |
| chr3L | 3957671 | 3957672 | CG12605 | 18.9 | 22.2 | 20.55 | chr3L_3957672_CG12605 |
| chr3L | 3961590 | 3961591 | CG12605 | 18.3 | 18.5 | 18.4 | chr3L_3961591_CG12605 |
| chr3L | 3992789 | 3992790 | scrt | 21.08 | 27.28 | 24.18 | chr3L_3992790_scrt |
| chr3L | 4092142 | 4092143 | CG14989 | 15.88 | 18.6 | 17.24 | chr3L_4092143_CG14989 |
| chr3L | 4113123 | 4113124 | Ack | 18.4 | 16.7 | 17.55 | chr3L_4113124_Ack |
| chr3L | 4113297 | 4113298 | Ack | 18.4 | 17.9 | 18.15 | chr3L_4113298_Ack |
| chr3L | 572527 | 572528 | hipk | 29.8 | 31 | 30.4 | chr3L_572528_hipk |
| chr3L | 572530 | 572531 | hipk | 42.7 | 43 | 42.85 | chr3L_572531_hipk |
| **chr3L** | **575712** | **575713** | **hipk** | **36.12** | **52.92** | **44.52** | **chr3L_575713_hipk** |
| **chr3L** | **575753** | **575754** | **hipk** | **31.7** | **49.6** | **40.65** | **chr3L_575754_hipk** |
| chr3L | 576730 | 576731 | hipk | 17.1 | 15.2 | 16.15 | chr3L_576731_hipk |
| chr3L | 577020 | 577021 | hipk | 20 | 16.9 | 18.45 | chr3L_577021_hipk |
| **chr3L** | **577417** | **577418** | **hipk** | **51.42** | **62.12** | **56.77** | **chr3L_577418_hipk** |
| chr3L | 577970 | 577971 | hipk | 21.4 | 25.9 | 23.65 | chr3L_577971_hipk |
| chr3L | 578186 | 578187 | hipk | 18.2 | 24.9 | 21.55 | chr3L_578187_hipk |
| chr3L | 578453 | 578454 | hipk | 25.7 | 32.7 | 29.2 | chr3L_578454_hipk |
| chr3L | 579335 | 579336 | hipk | 18.1 | 17 | 17.55 | chr3L_579336_hipk |
| chr3L | 579634 | 579635 | hipk | 20.5 | 19.8 | 20.15 | chr3L_579635_hipk |
| **chr3L** | **580103** | **580104** | **hipk** | **63.1** | **67.7** | **65.4** | **chr3L_580104_hipk** |
| chr3L | 580500 | 580501 | hipk | 15.6 | 18.1 | 16.85 | chr3L_580501_hipk |
| chr3L | 580932 | 580933 | hipk | 17.9 | 24.1 | 21 | chr3L_580933_hipk |
| **chr3L** | **8970787** | **8970788** | **CG5026** | **17.9** | **16.7** | **17.3** | **chr3L_8970788_CG5026** |
| chr3L | 8993382 | 8993383 | smg | 25.9 | 15.3 | 20.6 | chr3L_8993383_smg |
| chr3L | 9074752 | 9074753 | Tequila | 29.8 | 19.5 | 24.65 | chr3L_9074753_Tequila |
| **chr3L** | **9103274** | **9103275** | **bol** | **23.9** | **26.3** | **25.1** | **chr3L_9103275_bol** |
| chr3L | 9136454 | 9136455 | Use1 | 25 | 16 | 20.5 | chr3L_9136455_Use1 |
| chr3L | 9669496 | 9669497 | fry | 18.2 | 18.2 | 18.2 | chr3L_9669497_fry |
| chr3L | 9945905 | 9945906 | CG34356 | 19.4 | 32.1 | 25.75 | chr3L_9945906_CG34356 |
| chr3R | 10158991 | 10158992 | Invadolysin | 17.9 | 18.2 | 18.05 | chr3R_10158992_Invadolysin |
| chr3R | 10862820 | 10862821 | CG6574 | 20.8 | 33.3 | 27.05 | chr3R_10862821_CG6574 |
| chr3R | 10877257 | 10877258 | CR45195 | 32.7 | 19.5 | 26.1 | chr3R_10877258_CR45195 |
| chr3R | 10889941 | 10889942 | Leash | 18.2 | 18.8 | 18.5 | chr3R_10889942_Leash |
| **chr3R** | **13224637** | **13224638** | **Ace** | **24.8** | **33.3** | **29.05** | **chr3R_13224638_Ace** |
| **chr3R** | **13227870** | **13227871** | **Ace** | **26.6** | **35.3** | **30.95** | **chr3R_13227871_Ace** |
| **chr3R** | **13227970** | **13227971** | **Ace** | **33.42** | **47.95** | **40.69** | **chr3R_13227971_Ace** |
| chr3R | 14330745 | 14330746 | NK7.1 | 16.2 | 16.7 | 16.45 | chr3R_14330746_NK7.1 |
| chr3R | 14660839 | 14660840 | Hexim | 17.1 | 20 | 18.55 | chr3R_14660840_Hexim |
| chr3R | 14669984 | 14669985 | Meltrin | 16.95 | 20.7 | 18.82 | chr3R_14669985_Meltrin |
| chr3R | 14746335 | 14746336 | jvl | 36.4 | 20.9 | 28.65 | chr3R_14746336_jvl |
| chr3R | 14746546 | 14746547 | smp-30 | 45.1 | 24 | 34.55 | chr3R_14746547_smp-30 |
| **chr3R** | **14804349** | **14804350** | **btsz** | **16.5** | **26.5** | **21.5** | **chr3R_14804350_btsz** |
| **chr3R** | **15255687** | **15255688** | **CG42404** | **18.2** | **17.6** | **17.9** | **chr3R_15255688_CG42404** |
| chr3R | 15356475 | 15356476 | Atg4b | 15.4 | 20.7 | 18.05 | chr3R_15356476_Atg4b |
| chr3R | 15414730 | 15414731 | Atx2 | 22.82 | 26.25 | 24.54 | chr3R_15414731_Atx2 |
| chr3R | 15414731 | 15414732 | Atx2 | 19 | 22.6 | 20.8 | chr3R_15414732_Atx2 |
| chr3R | 15849417 | 15849418 | cv-d | 18.8 | 22.2 | 20.5 | chr3R_15849418_cv-d |
| **chr3R** | **16611267** | **16611268** | **NPF** | **22.98** | **23.52** | **23.25** | **chr3R_16611268_NPF** |
| chr3R | 16645743 | 16645744 | CG10324 | 26.1 | 35 | 30.55 | chr3R_16645744_CG10324 |
| chr3R | 17090737 | 17090738 | cal1 | 25 | 15.4 | 20.2 | chr3R_17090738_cal1 |
| **chr3R** | **17731884** | **17731885** | **Lgr1** | **20** | **19** | **19.5** | **chr3R_17731885_Lgr1** |
| chr3R | 17802763 | 17802764 | CG17806 | 20.7 | 18.8 | 19.75 | chr3R_17802764_CG17806 |
| chr3R | 19155252 | 19155253 | CG11779 | 15.6 | 19.1 | 17.35 | chr3R_19155253_CG11779 |
| chr3R | 20783186 | 20783187 | Syp | 21.1 | 17 | 19.05 | chr3R_20783187_Syp |
| chr3R | 20797469 | 20797470 | Syp | 15.3 | 20 | 17.65 | chr3R_20797470_Syp |
| chr3R | 20820785 | 20820786 | CG17271 | 31.2 | 19.75 | 25.48 | chr3R_20820786_CG17271 |
| chr3R | 20862212 | 20862213 | CG3822 | 17 | 26.3 | 21.65 | chr3R_20862213_CG3822 |
| chr3R | 20992668 | 20992669 | Calx | 17.8 | 27.5 | 22.65 | chr3R_20992669_Calx |
| chr3R | 21213891 | 21213892 | SNF4Agamm | 17.2 | 20 | 18.6 | chr3R_21213892_SNF4Agamm a |
|  |  |  | a |  |  |  |  |
| chr3R | 21354284 | 21354285 | mod(mdg4) | 15 | 19 | 17 | chr3R_21354285_mod(mdg4) |
| chr3R | 21527750 | 21527751 | CG7956 | 30.3 | 26.5 | 28.4 | chr3R_21527751_CG7956 |
| chr3R | 21625731 | 21625732 | E2f | 18 | 23.7 | 20.85 | chr3R_21625732_E2f |
| chr3R | 23261698 | 23261699 | orb | 15.2 | 16.1 | 15.65 | chr3R_23261699_orb |
| chr3R | 23681818 | 23681819 | eIF-3p66 | 20.4 | 21.5 | 20.95 | chr3R_23681819_eIF-3p66 |
| **chr3R** | **23698883** | **23698884** | **prt** | **15.4** | **23.4** | **19.4** | **chr3R_23698884_prt** |
| chr3R | 23723418 | 23723419 | CG10365 | 16.1 | 21.1 | 18.6 | chr3R_23723419_CG10365 |
| chr3R | 23732353 | 23732354 | Rpn9 | 20.4 | 16.7 | 18.55 | chr3R_23732354_Rpn9 |
| chr3R | 24664388 | 24664389 | slo | 23.1 | 30 | 26.55 | chr3R_24664389_slo |
| chr3R | 24802164 | 24802165 | polybromo | 29.2 | 20.98 | 25.09 | chr3R_24802165_polybromo |
| chr3R | 24820485 | 24820486 | Saf-B | 15 | 22 | 18.5 | chr3R_24820486_Saf-B |
| chr3R | 25234647 | 25234648 | CG10420 | 22.2 | 34.5 | 28.35 | chr3R_25234648_CG10420 |
| **chr3R** | **26233920** | **26233921** | **CG12290** | **25.6** | **18.4** | **22** | **chr3R_26233921_CG12290** |
| **chr3R** | **28050111** | **28050112** | **CG34362** | **15.6** | **25.6** | **20.6** | **chr3R_28050112_CG34362** |
| chr3R | 28838531 | 28838532 | Apc | 20.4 | 18.8 | 19.6 | chr3R_28838532_Apc |
| chr3R | 29659085 | 29659086 | Dop1R2 | 15.8 | 39.5 | 27.65 | chr3R_29659086_Dop1R2 |
| **chr3R** | **29674515** | **29674516** | **Bub3** | **27.6** | **17.8** | **22.7** | **chr3R_29674516_Bub3** |
| chr3R | 31457698 | 31457699 | Gprk2 | 23.5 | 24.5 | 24 | chr3R_31457699_Gprk2 |
| chr3R | 31841367 | 31841368 | RhoGAP100F | 17.73 | 19 | 18.37 | chr3R_31841368_RhoGAP100F |
| **chr3R** | **5811503** | **5811504** | **CG11000** | **21.3** | **32.4** | **26.85** | **chr3R_5811504_CG11000** |
| **chr3R** | **5811505** | **5811506** | **CG11000** | **20.8** | **25.7** | **23.25** | **chr3R_5811506_CG11000** |
| chr3R | 7126383 | 7126384 | CG10098 | 18.6 | 19.1 | 18.85 | chr3R_7126384_CG10098 |
| chr3R | 8244435 | 8244436 | CG18749 | 15.4 | 16.1 | 15.75 | chr3R_8244436_CG18749 |
| chr3R | 8244435 | 8244436 | CG33722 | 15.4 | 16.1 | 15.75 | chr3R_8244436_CG33722 |
| chr3R | 9416762 | 9416763 | alpha-Man-II | 30.8 | 32.3 | 31.55 | chr3R_9416763_alpha-Man-II |
| chr3R | 9441505 | 9441506 | ps | 37.92 | 33.52 | 35.72 | chr3R_9441506_ps |
| chr3R | 9471135 | 9471136 | CG16779 | 43.05 | 34.85 | 38.95 | chr3R_9471136_CG16779 |
| chr3R | 9525979 | 9525980 | CG8176 | 16.2 | 24.4 | 20.3 | chr3R_9525980_CG8176 |
| chr3R | 9539464 | 9539465 | mura | 21.75 | 20.05 | 20.9 | chr3R_9539465_mura |
| chr3R | 9794186 | 9794187 | CG8516 | 19.4 | 30.8 | 25.1 | chr3R_9794187_CG8516 |
| chr4 | 478956 | 478957 | Asator | 15.1 | 20 | 17.55 | chr4_478957_Asator |
| chr4 | 532906 | 532907 | zfh2 | 20 | 19.7 | 19.85 | chr4_532907_zfh2 |
| chr4 | 92946 | 92947 | pan | 22.5 | 20.8 | 21.65 | chr4_92947_pan |
| chrX | 10309093 | 10309094 | alpha-Man-I | 24.5 | 29 | 26.75 | chrX_10309094_alpha-Man-I |
| **chrX** | **12331302** | **12331303** | **Ten-a** | **34.8** | **34** | **34.4** | **chrX_12331303_Ten-a** |
| chrX | 16075472 | 16075473 | Tob | 23.68 | 29.32 | 26.5 | chrX_16075473_Tob |
| chrX | 16075887 | 16075888 | Tob | 22.2 | 22 | 22.1 | chrX_16075888_Tob |
| chrX | 16075888 | 16075889 | Tob | 21.6 | 24 | 22.8 | chrX_16075889_Tob |
| **chrX** | **16076959** | **16076960** | **Tob** | **20** | **23.3** | **21.65** | **chrX_16076960_Tob** |
| chrX | 16077193 | 16077194 | Tob | 17.85 | 19.15 | 18.5 | chrX_16077194_Tob |
| chrX | 16089317 | 16089318 | Tob | 15.2 | 16.1 | 15.65 | chrX_16089318_Tob |
| **chrX** | **3321068** | **3321069** | **dnc** | **22.7** | **28.9** | **25.8** | **chrX_3321069_dnc** |
| **chrX** | **3342369** | **3342370** | **dnc** | **17.4** | **22.2** | **19.8** | **chrX_3342370_dnc** |
| **chrX** | **6325433** | **6325434** | **CG15894** | **32** | **20.8** | **26.4** | **chrX_6325434_CG15894** |
| **chrX** | **9172798** | **9172799** | **mei-P26** | **17.3** | **23.1** | **20.2** | **chrX_9172799_mei-P26** |
| chrX | 9179452 | 9179453 | mei-P26 | 17.35 | 22.23 | 19.79 | chrX_9179453_mei-P26 |
| chrX | 9188891 | 9188892 | mei-P26 | 15.8 | 18.4 | 17.1 | chrX_9188892_mei-P26 |
