# Supplementary material for: Structured and disordered regions of Ataxin-2 contribute differently to the specificity and efficiency of mRNP granule formation
Source: PLoS Genet. 2024 May 20;20(5):e1011251. doi: 10.1371/journal.pgen.1011251 (PMC11166328; doi:10.1371/journal.pgen.1011251)
Supplement: S2 File — (DOCX) [file pgen.1011251.s002.docx]

# Table A: Key reagents used for the study.

| **Reagent type** **(species) or** **resource** | **Designation** | **Source or** **reference** | **Identifiers** | **Additional** **information** |
| --- | --- | --- | --- | --- |
| Genetic reagent (*Drosophila melanogaster*) | *UAS-Atx2-WTADARcd* | [1] | N/A |  |
| Genetic reagent (*Drosophila melanogaster*) | *UAS-Atx2-ΔLSmADARcd* | [1] | N/A |  |
| Genetic reagent (*Drosophila melanogaster*) | (*UAS-Atx2- ΔPAM2-*  *ADARcd*) | This paper | N/A |  |
| Genetic reagent (*Drosophila melanogaster*) | *UAS-Atx2- ΔcIDRADARcd* | [1] | N/A |  |
| Genetic reagent (*Drosophila melanogaster*) | *UAS-Atx2-only*  *LSm/Lsm-AD ADARcd* | [1] | N/A |  |
| Genetic reagent (*Drosophila melanogaster*) | *mef2-Gal4; TubGal80^ts^* | Bloomington *Drosophila* Stock Center | Derived from  #50742 |  |
| Genetic reagent (*Drosophila melanogaster*) | *elav-Gal4; TubGal80^ts^* | Bloomington *Drosophila* Stock Center | Derived from #458 |  |
| Cell line (Drosophila melanogaster) | S2R+ cells | DGRC | RRID:CVCL_Z831 |  |
| Cell line (Human) | HEK293T | Gift: Adrian Bracken lab | N/A |  |
| Cell line (Human) | U2OS | Gift: Martina Schroeder lab | N/A |  |
| Recombinant DNA  reagent | pUASt-Atx2-  SNAP_fly (Plasmid) | This paper | N/A | Construct to express fly  Atx2WT -SNAP |
| Recombinant DNA  reagent | pUASt-ATXN2-  SNAP_hum (Plasmid) | This paper | N/A | Construct to express human WT ATXN2-SNAP |
| Recombinant DNA  reagent | pUASt-mini-Atx2-  SNAP_fly (Plasmid) | This paper | N/A | Construct to express fly mini Atx2-SNAP |
| Recombinant DNA  reagent | pUASt-mini-ATXN2-  SNAP_hum (Plasmid) | This paper | N/A | Construct to express human mini ATXN2-SNAP |
| Recombinant DNA  reagent | pUASt-∆LSm-miniAtx2-SNAP_fly (Plasmid) | This paper | N/A | Construct to express fly ∆LSm mini Atx2-SNAP |
| Recombinant DNA  reagent | pUASt-∆LSm-miniATXN2-SNAP_hum  (Plasmid) | This paper | N/A | Construct to express human ∆LSm mini ATXN2-SNAP |
| Recombinant DNA  reagent | pUASt-∆LSmAD-  mini-Atx2-SNAP_fly (Plasmid) | This paper | N/A | Construct to express fly  ∆LSm-AD mini Atx2SNAP |
| Recombinant DNA  reagent | pUASt-∆LSmADmini-ATXN2-  SNAP_hum (Plasmid) | This paper | N/A | Construct to express human  ∆LSm-AD mini ATXN2SNAP |
| Recombinant DNA  reagent | pUASt-∆PAM2-miniAtx2-SNAP_fly (Plasmid) | This paper | N/A | Construct to express fly ∆PAM2 mini Atx2-SNAP |
| Recombinant DNA  reagent | pUASt-∆PAM2-miniATXN2-SNAP_hum  (Plasmid) | This paper | N/A | Construct to express human  ∆PAM2 mini ATXN2SNAP |

| Recombinant DNA  reagent | pUASt-L859A-miniAtx2-SNAP_fly  (Plasmid) | This paper | N/A | Construct to express fly PAM2* L859A mini Atx2SNAP |
| --- | --- | --- | --- | --- |
| Recombinant DNA  reagent | pUASt-L914A-miniATXN2-SNAP_hum  (Plasmid) | This paper | N/A | Construct to express human  PAM2* L914A mini  ATXN2-SNAP |
| Recombinant DNA  reagent | pUASt-F866A-mini-  Atx2-SNAP_fly  (Plasmid) | This paper | N/A | Construct to express fly PAM2* F866A mini Atx2SNAP |
| Recombinant DNA  reagent | pUASt-F921A-miniATXN2-SNAP_hum  (Plasmid) | This paper | N/A | Construct to express human  PAM2* F921A mini  ATXN2-SNAP |
| Recombinant DNA  reagent | pUASt-L859A-  F866A-mini-Atx2SNAP_fly (Plasmid) | This paper | N/A | Construct to express fly  PAM2* L859A and F866A mini Atx2-SNAP |
| Recombinant DNA  reagent | pUASt-L914AF921A-Q928A-miniATXN2-SNAP_hum  (Plasmid) | This paper | N/A | Construct to express human PAM2* L914A, F921A and Q928A mini ATXN2SNAP |
| Recombinant DNA  reagent | pAcman-Atx2-GFP (Fosmid) | [2] | N/A | Genomic construct to express fly WT Atx2-GFP |
| Recombinant DNA  reagent | pAcman-∆LSm-Atx2GFP (Fosmid) | This paper | N/A | Genomic construct to  express fly ∆LSm Atx2GFP |
| Recombinant DNA  reagent | pAcman-∆PAM2-  Atx2-GFP (Fosmid) | This paper | N/A | Genomic construct to  express fly ∆PAM2 Atx2GFP |
| Recombinant DNA  reagent | pActin-Gal4 | DGRC |  | Actin promoter Gal4 for insect UAS expression |
| Recombinant DNA  reagent | pCMV-Gal4 | Addgene | #24345 | CMV promoter Gal4 for  mammalian UAS  expression |
| Antibody | Anti-Atx2 (chicken polyclonal) | [3] | N/A | IF (1:1000) |
|  |  |  |  | WB (1:1000) |
| Antibody | Anti-Caprin (rabbit polyclonal) | [4] | N/A | IF (1:1000) |
| Antibody | Anti-dFMR (mouse monoclonal) | DSHB | # 5A11 | IF (1:1000) deposited to the DSHB by Siomi, H. |
| Antibody | Anti-GFP (chicken polyclonal) | Abcam | Cat# mAb 13970 | IF (1:1000) |
| Antibody | Anti-V5 (rabbit polyclonal) | Santa Cruz  Biotechnology | Cat# sc83849-R | IF (1:1000) |
|  |  |  |  | WB (1:1000) |
| Antibody | PABP (rabbit polyclonal serum) | [5] | N/A | IF (1:500) WB (1:500) |
| Antibody | Me31B (rabbit polyclonal serum) |  | N/A | IF (1:500) |
| Antibody | Rox8 (rat polyclonal) | [6] | N/A | IF (1:1000) |
| Antibody | SNAP (rabbit polyclonal) | NEB | Cat# P9310S | WB (1:1000) |
| Antibody | PABPC1 (rabbit polyclonal) | Abcam | Cat# ab21060 | WB (1:1000) |
| Antibody | LSM12 (rabbit anti human polyclonal) | Abcam | Cat# ab173292 | WB (1:1000) |
| Antibody | G3BP1 (mouse monoclonal) | BD Bioscience | Cat# 611126 | IF (1:1000) |
| Antibody | Histone H3 (rabbit polyclonal) | Cell signaling tech | Cat# 9715 | WB (1:1000) |
| Antibody | Baf155 (rabbit monoclonal) | Cell signaling tech | Cat# 11956 | WB (1:1000) |

| Antibody | HRP Goat anti-rabbit | Invitrogen | Cat# A16104 | WB (1:10,000) |
| --- | --- | --- | --- | --- |
| Antibody | HRP Goat anti-mouse | Invitrogen | Cat# 31430 | WB (1:10,000) |
| Antibody | Alexa Fluor 555 (polyclonal goat antichicken IgG) | Invitrogen | Cat# A21437 | IF (1:1000) |
| Antibody | Alexa Fluor 488 (polyclonal goat antichicken IgG) | Invitrogen | Cat# A11039 | IF (1:1000) |
| Antibody | Alexa Fluor 647 (polyclonal goat antichicken IgG) | Invitrogen | Cat# A21449 | IF (1:1000) |
| Antibody | Alexa Fluor 555 (polyclonal goat antirabbit IgG) | Invitrogen | Cat# A21428 | IF (1:1000) |
| Antibody | Alexa Fluor 488 (polyclonal goat antirabbit IgG) | Invitrogen | Cat# A11078 | IF (1:1000) |
| Antibody | Alexa Fluor 647 (polyclonal goat antirabbit IgG) | Invitrogen | Cat# A21244 | IF (1:1000) |
| Antibody | Alexa Fluor 555  (polyclonal goat antimouse IgG) | Invitrogen | Cat# A21422 | IF (1:1000) |
| Antibody | Alexa Fluor 488 | Invitrogen | Cat# A21121 | IF (1:1000) |
|  | (polyclonal goat antimouse IgG) |  |  |  |
| Antibody | Alexa Fluor 647  (polyclonal goat antimouse IgG) | Invitrogen | Cat# A21235 | IF (1:1000) |
| Chemical compound | MOWIOL mounting medium | Sigma (Merck) | Cat# 81381 |  |
| Chemical compound | SNAP-TmrStar | New England Biolabs | Cat# S9105S | IF (1:1000) |
| Chemical compound | SNAP-Surface 488 | New England Biolabs | Cat# S9124S | IF (1:1000) |
| Software, algorithm | TRIBE | [7] | https://github.com/ro sbashlab/TRIBE |  |
| Software, algorithm | STAR v2.5.3 | [8] | https://github.com/ale xdobin/STAR |  |
| Software, algorithm | HTSeq v0.11.2 | [9] | https://github.com/htseq/htseq |  |
| Software, algorithm | DESeq2 | [10] | https://bioconductor. org/packages/release/ bioc/html/DESeq2.ht ml |  |
| Software, algorithm | Guitar | [11] | https://bioconductor. org/packages/release/ bioc/html/Guitar.html |  |
| Software, algorithm | Bedtools | [12] | https://github.com/ar q5x/bedtools2 |  |
| Software, algorithm | twoBitToFa | - | https://genome.ucsc.edu/  goldenPath/help/twoBit.html |  |
| Software, algorithm | ImageJ/Fiji | [13] | https://imagej.nih.gov/ij/ |  |
| Software, algorithm | Ggplot2 | [14] | https://github.com/tid yverse/ggplot2 |  |
| Software, algorithm | Pheatmap |  | https://cran.rproject.org/web/pack ages/pheatmap/index.  html |  |
| Software, algorithm | SnapDragon |  | https://www.flyrnai.o rg/snapdragon |  |

References:

1. Singh A, Hulsmeier J, Kandi AR, Pothapragada SS, Hillebrand J, Petrauskas A, et al. Antagonistic roles for Ataxin-2 structured and disordered domains in RNP condensation. 2021;2: 1–26.

2. Sudhakaran IP, Hillebrand J, Dervan A, Das S, Holohan EE, Hülsmeier J, et al. FMRP and Ataxin-2 function together in long-term olfactory habituation and neuronal translational control. Proc Natl Acad Sci U S A. 2014;111: E99–E108. doi:10.1073/pnas.1309543111

3. Bakthavachalu B, Huelsmeier J, Sudhakaran IP, Hillebrand J, Singh A, Petrauskas A, et al. RNP-Granule Assembly via Ataxin-2 Disordered Domains Is Required for Long-Term Memory and Neurodegeneration. Neuron. 2018;98: 754-766.e4. doi:10.1016/j.neuron.2018.04.032

4. Papoulas O, Monzo KF, Cantin GT, Ruse C, Yates JR, Ryu YH, et al. dFMRP and Caprin , translational regulators of synaptic plasticity , control the cell cycle at the Drosophila mid-blastula transition. 2010;4209: 4201–4209. doi:10.1242/dev.055046

5. Lee J, Yoo E, Lee H, Park K, Hur JH, Lim C. LSM12 and ME31B/DDX6 Define Distinct Modes of Posttranscriptional Regulation by ATAXIN-2 Protein Complex in Drosophila Circadian Pacemaker Neurons. Mol Cell. 2017;66: 129-140.e7. doi:10.1016/j.molcel.2017.03.004

6. Buddika K, Ariyapala IS, Hazuga MA, Riffert D, Sokol NS. Canonical nucleators are dispensable for stress granule assembly in Drosophila intestinal progenitors. J Cell Sci. 2020;133. doi:10.1242/jcs.243451

7. McMahon AC, Rahman R, Jin H, Shen JL, Fieldsend A, Luo W, et al. TRIBE: Hijacking an RNA-Editing Enzyme to Identify Cell-Specific Targets of RNA-Binding Proteins. Cell. 2016;165: 742–753. doi:10.1016/j.cell.2016.03.007

8. Dobin A, Davis CA, Schlesinger F, Drenkow J, Zaleski C, Jha S, et al. STAR: ultrafast universal RNA-seq aligner. Bioinformatics. 2013;29: 15–21. doi:10.1093/bioinformatics/bts635

9. Anders S, Pyl PT, Huber W. HTSeq--a Python framework to work with high-throughput sequencing data. Bioinformatics. 2015;31: 166–169. doi:10.1093/bioinformatics/btu638

10. Love MI, Huber W, Anders S. Moderated estimation of fold change and dispersion for RNA-seq data with DESeq2. Genome Biol. 2014;15: 550. doi:10.1186/s13059-014-0550-8

11. Cui X, Wei Z, Zhang L, Liu H, Sun L, Zhang S-W, et al. Guitar: An R/Bioconductor Package for Gene Annotation Guided Transcriptomic Analysis of RNA-Related Genomic Features. Biomed Res Int. 2016;2016: 8367534. doi:10.1155/2016/8367534

12. Quinlan AR, Hall IM. BEDTools: a flexible suite of utilities for comparing genomic features. Bioinformatics. 2010;26: 841–842. doi:10.1093/bioinformatics/btq033

13. Schindelin J, Arganda-Carreras I, Frise E, Kaynig V, Longair M, Pietzsch T, et al. Fiji: an open-source platform for biological-image analysis. Nat Methods. 2012;9: 676–682. doi:10.1038/nmeth.2019

14. Wilkinson L. ggplot2: Elegant Graphics for Data Analysis by WICKHAM, H. Biometrics. 2011;67: 678–679. doi:10.1111/j.1541-0420.2011.01616.x
